# Post-vaccine epidemiology of serotype 3 pneumococci identifies transformation inhibition through prophage-driven alteration of a non-coding RNA

**DOI:** 10.1186/s13073-022-01147-2

**Published:** 2022-12-20

**Authors:** Min Jung Kwun, Alexandru V. Ion, Hsueh-Chien Cheng, Joshua C. D’Aeth, Sam Dougan, Marco R. Oggioni, David A. Goulding, Stephen D. Bentley, Nicholas J. Croucher

**Affiliations:** 1 MRC Centre for Global Infectious Disease Analysis, Department of Infectious Disease Epidemiology, School of Public Health, White City Campus, Imperial College London, London, W12 0BZ UK; 2 Parasites & Microbes, Wellcome Sanger Institute, Wellcome Genome Campus, Hinxton, Cambridge, CB10 1SA UK; 3 Department of Genetics, University of Leicester, University Road, Leicester, LE1 7RH UK; 4 Dipartimento di Farmacia e Biotecnologie, Università di Bologna, Via Irnerio 42, 40126 Bologna, Italy

**Keywords:** Genomic epidemiology, Pneumococcus, Vaccine, Prophage, Transformation, Recombination, Non-coding RNA, Selfish DNA

## Abstract

**Background:**

The respiratory pathogen *Streptococcus pneumoniae* (the pneumococcus) is a genetically diverse bacterium associated with over 101 immunologically distinct polysaccharide capsules (serotypes). Polysaccharide conjugate vaccines (PCVs) have successfully eliminated multiple targeted serotypes, yet the mucoid serotype 3 has persisted despite its inclusion in PCV13. This capsule type is predominantly associated with a single globally disseminated strain, GPSC12 (clonal complex 180).

**Methods:**

A genomic epidemiology study combined previous surveillance datasets of serotype 3 pneumococci to analyse the population structure, dynamics, and differences in rates of diversification within GPSC12 during the period of PCV introductions. Transcriptomic analyses, whole genome sequencing, mutagenesis, and electron microscopy were used to characterise the phenotypic impact of loci hypothesised to affect this strain’s evolution.

**Results:**

GPSC12 was split into clades by a genomic analysis. Clade I, the most common, rarely underwent transformation, but was typically infected with the prophage ϕOXC141. Prior to the introduction of PCV13, this clade’s composition shifted towards a ϕOXC141-negative subpopulation in a systematically sampled UK collection. In the post-PCV13 era, more rapidly recombining non-Clade I isolates, also ϕOXC141-negative, have risen in prevalence. The low in vitro transformation efficiency of a Clade I isolate could not be fully explained by the ~100-fold reduction attributable to the serotype 3 capsule. Accordingly, prophage ϕOXC141 was found to modify csRNA3, a non-coding RNA that inhibits the induction of transformation. This alteration was identified in ~30% of all pneumococci and was particularly common in the unusually clonal serotype 1 GPSC2 strain. RNA-seq and quantitative reverse transcriptase PCR experiments using a genetically tractable pneumococcus demonstrated the altered csRNA3 was more effective at inhibiting production of the competence-stimulating peptide pheromone. This resulted in a reduction in the induction of competence for transformation.

**Conclusion:**

This interference with the quorum sensing needed to induce competence reduces the risk of the prophage being deleted by homologous recombination. Hence the selfish prophage-driven alteration of a regulatory RNA limits cell-cell communication and horizontal gene transfer, complicating the interpretation of post-vaccine population dynamics.

**Supplementary Information:**

The online version contains supplementary material available at 10.1186/s13073-022-01147-2.

## Background

*Streptococcus pneumoniae* (the pneumococcus) is a globally endemic gram-positive nasopharyngeal commensal bacterium and respiratory pathogen that causes both common (e.g., conjunctivitis, otitis media) and invasive (e.g., sepsis and meningitis) infections [1]. The species is genetically diverse and has been subdivided into hundreds of Global Pneumococcal Sequencing Clusters (GPSCs) by the Global Pneumococcal Sequencing (GPS) project [2, 3]. These GPSCs, which can be described as distinct strains, encode characteristic combinations of accessory loci from the pneumococcus’ extensive pangenome [4, 5]. These can be exchanged between isolates through two classes of mechanism. The first is the movement of mobile genetic elements (MGEs), commonly represented by prophage, phage-related chromosomal islands (PRCIs), and integrative and conjugative elements (ICEs) in pneumococci [4]. The second is cell-driven transformation, which integrates exogenous DNA into the chromosome through homologous recombination [6]. This favours deletion of accessory loci, including MGEs, over insertion [7]. Therefore, the two types of mechanism can be viewed as making conflicting contributions to bacterial evolution [8].

Transformation enables exchange of genetic variation between strains across the chromosome, including the highly diverse capsule polysaccharide synthesis (*cps*) locus [9]. This encodes the machinery for generating the polysaccharide capsule and therefore determines an isolate’s serotype [10]. Over 101 immunologically distinct serotypes are expressed by pneumococci [11, 12], which are associated with variation in carriage duration [13] and propensity to cause invasive disease [14]. Most capsules are covalently attached to the peptidoglycan cell wall by Wzg (or Cps2A) enzymes [10]. The exceptions are the “mucoid” capsules 3 and 37, which are instead anchored to the membrane through attachment to phosphatidylglycerol [15]. Their mucoid appearance results from polymerases that operate continuously until synthesis is stopped by low substrate concentrations, causing the serotypes’ distinctive large colony morphologies [16].

The burden of pneumococcal disease motivated the development of polysaccharide conjugate vaccines (PCVs) that target a subset of serotypes [17]. These formulations comprise polysaccharides attached to carrier proteins, enabling infants to mount an effective adaptive immune response to the capsule antigens [18]. The increased antigenicity of protein-associated polysaccharides was first demonstrated with the serotype 3 capsule, which was attached to a horse serum globulin and used to induce protective immunity in rabbits in the 1930s [19]. Yet the first national immunisation campaign with a PCV targeting pneumococci was not initiated until 2000 [1]. This initial 7-valent design (PCV7) was later expanded as PCV10 and PCV13 formulations, which were introduced in 2010 [17] and are currently used in 148 countries [20].

PCV13 was the first to include serotype 3, which was added due to it commonly causing invasive disease associated with a high mortality rate [21]. Although PCV13 has proved highly effective in eliminating other vaccine-targeted serotypes [17], serotype 3 infections have persisted, with absolute incidences not substantially altered from the pre-PCV era [22]. This has meant serotype 3 remains a major cause of invasive disease in many countries, particularly in adults [22, 23]. The poor effectiveness against serotype 3 [24] is likely to reflect both the relatively low immunogenicity of this component of PCV13 [25] (and the 11-valent predecessor of PCV10 [26]), and the shedding of serotype 3 capsule polysaccharides from the pneumococcal membrane, which inhibits antibody-mediated killing of these bacteria [27].

Despite the absence of substantial PCV13-associated population dynamics at the serotype level, genomic epidemiology has revealed contemporaneous changes within the serotype 3 pneumococcal population. Although it is one of the most common serotypes in a genetically diverse pathogen, the serotype 3 capsule is predominantly associated with just a small number of strains [3]: of the 887 serotype 3 *S. pneumoniae* genomes in Pathogenwatch, 566 (63.8%) belong to GPSC12 (equivalent to multi-locus sequence type clonal complex 180) [28]. Pre-PCV13, the European and North American population of GPSC12 was dominated by a genotype termed Clade I [29–31]. Representatives of this genetically homogeneous clade typically shared an unusually stable prophage, ϕOXC141 [29] (as known as ϕSpn_OXC [32]). There was also little variation in the rest of the chromosome, as the interstrain exchange of sequence through transformation was barely detectable in these bacteria [33]. Genomic analyses of other pneumococcal strains (GPSC23, or PMEN2 [34]; and GPSC18, or PMEN9 [35]) have identified similar non-recombining lineages. In these pneumococci, the absence of diversification was attributable to stably integrated MGEs that had disrupted competence genes required for transformation. These lineages underwent rapid local dissemination, prior to elimination by vaccine-induced immunity, as *cps* locus diversification was not observed in the absence of recombination. Analogously, in recent years, there has been a replacement of Clade I by non-Clade I serotype 3 GPSC12 isolates [30, 31]. However, phenotypic assays of isolates from the USA did not reveal any change in capsule expression that could directly link these changes to PCV-induced immunity [30], and the competence genes appear intact in Clade I [29, 30]. Therefore, we undertook a genomic analysis of the GPSC12 population to understand whether its post-PCV dynamics were consistent with the selection against low recombination genotypes observed in other pneumococcal strains, and whether the stably integrated MGE ϕOXC141 contributed to the variation in transformation rates.

## Methods

### Phylogenetic analysis of GPSC12

For the analysis of GPSC12’s population structure, an overlapping set of 1116 short-read datasets was collated from Azarian et al. (295 isolates mainly from Europe and the USA) [30], Groves et al. (616 isolates collected by Public Health England) [31], and the Global Pneumococcal Sequencing (GPS) project (205 isolates from https://microreact.org/project/gpsGPSC12) [36]. These datasets yielded a non-redundant set of 979 isolates, one of which (accession code ERR433970) was inaccessible. The short-read data for the remaining 978 isolates were assembled de novo with SPAdes version 3.10.1 [37] using default settings and k-mer lengths between 21 and 85 with a step size of four. Assemblies were evaluated with assembly-stats [38]. Seven sequences with anomalous assembly lengths, below 1.9 Mb or above 2.5 Mb, were removed (Additional file 1: Fig. S1).

The 971 remaining isolates assembled from short-read data, and six high-quality draft genomes from Croucher et al. [29], were assigned to strains using PopPUNK version 2.4.0 [2] and version 6 of the GPS project’s GPSC database [3]. This found 891 of the isolates belonged to strain GPSC12 and therefore were sufficiently closely related for phylogenetic analysis (accession codes listed in Additional file 2: Table S1).

The complete genome of GPSC12 isolate *S. pneumoniae* OXC141 (accession code FQ312027) was used as the reference genome against which all other 890 GPSC12 assemblies were mapped using SKA version 1.0 with default settings [39]. The resulting alignment of 891 whole genomes was analysed with Gubbins version 3.2.1 [40], using RapidNJ [41] and a Jukes-Cantor model to generate the original tree, with four subsequent iterations using RAxML [42] trees constructed with a General Time-Reversible model of base substitution using a gamma model of between-site rate heterogeneity (recombination statistics listed in Additional file 3: Table S2). Results were visualised with Phandango [36] and RCandy [43].

### Analysis of csRNA

Identification of csRNA used the corresponding Rfam model (accession code RF02379) [44]. Assembled genomes were scanned using the cmsearch tool from version 1.1.2 of the Infernal package [45]. Heuristic filters were set at the levels used for Rfam, and both significant and questionable hits were retained, to explore the full diversity of csRNA sequences.

To analyse the diversity of csRNA sequences in the GPS dataset, the 104,934 csRNA sequences were processed to identify a non-redundant set of 388 sequences that were all longer than 80 nt and contained no ambiguous bases. These were aligned with mafft v7.505 [46] using the default progressive alignment FFT-NS-2 method with a DNA200 model. A phylogeny was generated using FastTree version 2.1.11 [47] with a Jukes-Cantor model and a CAT approximation of between-site rate heterogeneity with 20 rate categories. The phylogeny was manually annotated with FigTree [48] and ggtree [49].

RNA structures were visualised using the minimum free energy prediction [50] generated by the RNAfold WebServer [51] with default settings, using the sequences listed in Additional file 4: Table S3. Structures were plotted using the R package RRNA [52]. Inference of the strength of interactions between csRNAs and the *comC* mRNA, using a previously identified *comC* transcription start site [53], used IntaRNA version 2.0 [54].

### Analysis of the accessory genome

The 871 assembled GPSC12 isolates were used to generate a sequence database with BLAST version 2.12.0 [55]. To identify intact *attB*_OXC_ sequences, a 4477-bp locus was extracted from the *S. pneumoniae* TIGR4 genome (accession code AE005672) [56], extending from the 5′ end of *purA* to the 3′ end of *radA*. GPSC12 isolates with a BLASTN match longer than 3750 bp were inferred to have an intact *attB*_OXC_ site, whereas isolates with only shorter alignments to the query sequence were inferred to have a prophage insertion at this site. Similarly, the presence of prophage ϕOXC141 was inferred through querying the database with the 34,080 bp ϕOXC141 sequence from *S. pneumoniae* OXC141 [29]. If the longest BLASTN hit was longer than 12,500 bp, then an isolate was inferred to be infected with ϕOXC141, or a very closely related prophage.

Phylodynamic analysis of Clade I applied BactDating v1.1.1 [57] to the subset of 638 isolates with a date of isolation, which ranged from 1993 to 2018. The analysis used a relaxed clock model, fitted using Markov chain Monte Carlo (MCMC) sampling run for 5×10^7^ iterations. Half of the chain was discarded as burn-in, with convergence of the second half of established through visual assessment of the MCMC chains. To estimate the date at which the ϕOXC141-negative subclade emerged, the presence and absence of ϕOXC141 was reconstructed over the internal nodes of the Gubbins phylogeny as a discrete state using PastML with the JOINT model [58].

To explain the absence of csRNA1 sequences from some of the GPS collection, the 20,047 sequences described by Gladstone et al. were used to generate a species-wide sequence database [3]. This was queried with the intergenic sequence upstream of *ruvB* in *S. pneumoniae* R6 (accession code AE007317) [59], the locus within which *ccnA* and *ccnB* were originally identified [60]. Assemblies with a BLAST alignment to the query that was longer than 900 bp were inferred to have a full-length locus encoding both *ccnA* and *ccnB*, whereas assemblies with an alignment below this threshold were assumed to have the chimeric gene *ccnAB*.

Pairwise alignments of genomes for analysis of specific accessory loci used BLASTN and were visualised using the Artemis Comparison Tool [61] and the R package genoPlotR [62].

### Culturing and passage of *S. pneumoniae*

*S. pneumoniae* were grown statically at 35 °C in 5% CO_2_, unless otherwise stated. Genotypes used in this study are listed in Additional file 5: Table S4. Liquid cultures were grown in a 2:3 ratio mixture of Todd-Hewitt media with 0.5% yeast extract (Sigma-Aldrich), and Brain-Heart Infusion media (Sigma-Aldrich), dissolved in milliQ (18MΩ) water, henceforth referred to as mixed media [63]. Culturing on solid media used Todd-Hewitt media supplemented with 0.5% yeast extract.

*S. pneumoniae* 99-4038 was passaged with the aim of isolating a ΔϕOXC141 mutant. For each round of the passage, an overnight culture was diluted 1:9 in fresh mixed media supplemented with 40 μg mL^−1^ catalase to a total volume of 10 mL. At an OD_600_ of between 0.3 and 0.4, mitomycin C was added to a final concentration of 0.1 μg mL^−1^. After three such passages, 24 colonies were picked and screened for the loss of ϕOXC141 by PCR amplification of the *attL*_OXC_ and *attB*_OXC_ sequences.

### Extraction of DNA and PCR amplification

Cultures were centrifuged for 10 min at 4000*g*, and the supernatant was discarded. Bacterial cell pellets were resuspended in 480 μL lysis buffer (Promega) and 120 μL 30 mg mL^−1^ lysozyme (Promega), followed by 30 min incubation at 35 °C. Samples were centrifuged at 8000*g* for 2 min, and DNA extracted from the pellets using the Wizard Genomic DNA Purification Kit (Promega) according to the manufacturer’s instructions.

Extraction of genomic DNA from *S. pneumoniae* R6 *cps*_99-4038_ for sequencing with Oxford Nanopore Technology used a different approach, to reduce the polysaccharide contamination of the final sample used for library preparation. A cell pellet was resuspended in 250 μL Tris-EDTA buffer and 50 μL 30 g L^−1^ lysozyme (Roche) in Tris-EDTA buffer. This mixture was vortexed at room temperature for 15 min and 400 μL 0.1 M EDTA (Gibco) and 250 μL 10% sarkosyl (BDH) were added. Samples were incubated at 4 °C for 2 h, prior to the addition of 50 μL proteinase K (Roche), 30 μl RNase A (Roche) and 3 mL Tris-EDTA buffer. Samples were incubated at 50 °C overnight, then washed with 5 mL of a 25:24:1 mixture of phenol, chloroform and indole-3-acetic acid (Fluka) and centrifuged (2594*g*, 10 min). The aqueous phase was removed, washed with 5 mL chloroform (Sigma-Aldrich) and centrifuged (2594*g*, 10 min). DNA was precipitated from the aqueous phase using 300% by volume ethanol and 10% by volume 3 M sodium acetate followed by 1 h incubation at −20 °C. The pellet recovered following centrifugation was then washed with 5 mL 70% ethanol and resuspended in water.

PCR amplification used 500 ng of template DNA, 7.5 μL 2× DreamTaq Master Mix (Thermo Fisher), nuclease-free water (Thermo Fisher) and 1 μl of a 10 μM solution of each of the forward and reverse primers. Primer sequences are listed in Additional file 6: Table S5.

To purify individual DNA amplicons, PCR products were separated using 1% agarose gels (Sigma-Aldrich) dyed with SYBR Safe (Invitrogen) in TBE buffer (Invitrogen) with a 1-kb HyperLadder (Bioline) marker. Where necessary, individual amplicons were excised and extracted with the GenElute Gel Extraction Kit (Sigma-Aldrich) according to the manufacturer’s instructions.

### Extraction and processing of RNA samples

To generate samples for RNA-seq, the four genotypes being analysed (*S. pneumoniae* RMV8_domi_, RMV8_domi_*tvr*::*cat*, RMV8_rare_ and RMV8_rare_*tvr*::*cat*) were grown as 500-μL cultures in a 48-well plate to an OD_600_ of 0.5. Four wells were then harvested at the appropriate growth stage and combined with 4 mL RNAprotect (Qiagen) to generate each replicate. These samples were then processed with the SV Total RNA Isolation System (Promega) according to the manufacturer’s instructions. The generation of cDNA and Illumina sequencing libraries was undertaken as described previously [63]. Three replicates for each genotype were sequenced as 200 nt paired-end multiplexed libraries on a single Illumina HiSeq 4000 lane.

To generate samples for qRT-PCR, cells were grown to the specified optical density in 10 mL mixed liquid media with 50 μL of 500 mM calcium chloride. A 5-mL sample was then mixed with 10 mL of RNAprotect and processed with the SV Total RNA Isolation System (Promega) according to the manufacturer’s instructions. DNA was removed from 0.5-μg samples of RNA with Amplification-grade DNAse I (Sigma-Aldrich). RNA was used to generate complementary DNA (cDNA) with the First-Strand III cDNA synthesis kit (Invitrogen). Amplification reactions used the PowerUp™ SYBR™ Green Master Mix (Thermo Fisher) and the QuantStudio™ 7 Flex Real-Time PCR System (Applied Biosystems).

### Analysis of RNA-seq data

The assembly of *S. pneumoniae* RMV8_rare_ (accession code OX244288) [64] was annotated with Prokka [65]. RNA-seq data (accession codes in Additional file 7: Table S6) were mapped to the 2132 annotated protein coding sequences with Kallisto version 0.46.2 [66] and analysed with Sleuth version 0.30.0 [67]. Gene expression levels were quantified as scaled reads per base, the normalised mean number of reads mapping to each position within a gene, which corrects for variation in gene length and the number of reads in each dataset. The patterns of gene transcription in *S. pneumoniae* RMV8_domi_ were used as the reference against which transcription in the other three genotypes (RMV8_rare_, RMV8_domi_*tvr*::*cat* and RMV8_rare_*tvr*::*cat*) were compared using Wald tests. The threshold for significant differences in transcription was set at a false discovery rate of 10^−3^ using Q-Q plots. Data were plotted using the R package circlize [68].

To calculate coverage of each strand of the genome, RNA-seq data were mapped to the whole RMV8_rare_ chromosome with BWA version 0.5.9 [69]. The resulting SAM file was edited by identifying all reverse reads that mapped in a proper pair, and inverting the strand to which they aligned. The coverage of each strand of the genome was then calculated using SAMtools version 1.9 [70].

### Analysis of quantitative PCR data

The ΔΔCt method was used to quantify gene expression through qRT-PCR. The *rpoA* gene was used as a reference gene in each sample, against which the expression of the gene of interest was normalised as a ΔCt value. In all experiments, three technical measurements were recorded for each biological replicate. The mean ΔCt for a selected standard gene in a particular sample was then calculated across technical replicates. This was used to calculate the ΔΔCt values for other genes within the same biological replicate. The fold difference between genotypes was then quantified as 2^−ΔΔCt^. Where possible, RMV8_rare_ was used as the standard sample.

For the quantification of prophage dynamics, the products of primers A and B (“AB”) and A and D (“AD”) were generated from genomic DNA, and their concentrations measured using the Qubit Broad Range kit and a Qubit 4 Fluorometer (Thermo Fisher). The Ct values for known concentrations of these products were used to generate a standard curve, which was used to convert Ct values from experiments into absolute copy numbers of DNA molecules.

### Assaying transformation efficiency

For assaying spontaneous transformation efficiency, 2×10^5^ cells of RMV8_rare_ were grown in 1 mL mixed media using a 12-well plate incubated at 35 °C, in a 5% CO_2_ atmosphere, with 5 μL 500 mM CaCl_2_ and 1.5 μg genomic DNA containing a rifampicin resistance marker. The same method was used to assay induced competence, except that 2.5 μL 0.5 mg mL^−1^ of the appropriate competence-stimulating peptide (CSP) was also added to the cell culture. For assays of DNase sensitivity, 2.5 units of DNase I (Thermo Fisher) was added to the cell culture. After 18 h of incubation, cells were transferred to solid selective media supplemented with 4 μg mL^−1^ rifampicin (Fisher Scientific). In parallel, titrations on non-selective plates were used to calculate the overall number of viable cells.

### Construction of mutants

The disruption of genes was achieved through amplifying the flanking ~1 kb regions with primers that added restriction sites on the internal sides of each. An antibiotic resistance cassette was then amplified with the corresponding restriction sites on either side. PCR amplicons were purified following gel electrophoresis as described above, then digested with the appropriate restriction enzyme (*Bam*HI or *Eco*RV; Promega). Both flanking regions were ligated to the resistance marker using T4 DNA ligase (Invitrogen).

To transform recipient bacteria with these constructs, *S. pneumoniae* was grown statically in mixed media to an OD_600_ of 0.2–0.25. A 1 mL sample of the bacterial culture was mixed with 5 μL 500 mM CaCl_2_ (Sigma-Aldrich), 2.5 μL of the appropriate competence-stimulating peptide (Sigma-Aldrich) and the PCR-amplified DNA construct. Tubes were incubated at 35 °C in 5% CO_2_ for 2 h prior to transfer onto solid media.

Transformants were selected on solid media supplemented with the appropriate antibiotic. When using a chloramphenicol acetyltransferase (*cat*) marker, media were supplemented with chloramphenicol (Sigma-Aldrich) at 4 μg mL^−1^. When selecting for isolates carrying the selectable and counter-selectable Janus cassette, media were supplemented with kanamycin (Sigma-Aldrich) at 600 μg mL^−1^; when selecting for isolates lacking the cassette, media were supplemented with streptomycin (Sigma-Aldrich) at 200 μg mL^−1^.

The Janus cassette was used to remove the *cps* locus of R6 Δ*ivr* to generate *S. pneumoniae* R6 Δ*ivr cps*::Janus. This intermediate genotype was transformed with genomic DNA from *S. pneumoniae* 99-4038, followed by selection on streptomycin, to identify bacteria in which the Janus cassette had been lost. Those bacteria which had replaced the cassette with the *cps* locus of 99-4038 were further screened for expression of the capsule, based on their colony morphology.

A modified version of the Janus cassette [71] was constructed in which *rpsL*, causing sensitivity to streptomycin, was replaced with a pheS* sequence, which caused sensitivity to *p*-chloro-phenylalanine (*p*-Cl-Phe) [72]. This Janus_*pheS*_ cassette enabled more effective counter-selection against bacteria carrying the cassette using *p*-Cl-Phe (VWR) at 3 μg mL^−1^. For the construction of RMV8_rare_ and RMV8_domi_ genotypes stably expressing *ccnC* or *ccnCL*, the ϕRMV8 prophage and adjacent *ccnCL* gene were replaced with this modified Janus cassette to generate ϕRMV8::Janus_*pheS*_ mutants. To restore the original *ccnC* sequence, the relevant locus was directly amplified from *S. pneumoniae* R6. To introduce the modified *ccnCL* without the associated prophage, the *ccnCL* sequence from RMV8 was amplified and ligated to the bacterial sequence flanking the ϕRMV8 *attR* site. Both resulting DNA amplicons were used to transform an R6 *attB*_OXC_::Janus_*pheS*_ mutant. Genomic DNA from these intermediate genotypes was then used to transform the ϕRMV8::Janus_*pheS*_ mutants, with selection on *p*-Cl-Phe used to identify recombinants that had replaced the Janus_*pheS*_ cassette. Dideoxy terminator sequencing was used to confirm the acquisition of the relevant *ccnC* and *ccnCL* alleles.

### Characterisation of the *S. pneumoniae* R6 *cps*_99-4038_ recombinant

Sequencing libraries were generated with the Oxford Nanopore Rapid Barcoding Sequencing Kit (Oxford Nanopore Technologies, code SQK-RBK004), according to the manufacturer’s instructions. Sequencing used a SpotON flow cell installed on a MinION device, generating 304 Mb of data, with a maximum read length of 63,258 bp. Bases were recalled with the high accuracy model of guppy version 6.0.1. Reads were assembled with dragonflye version 1.0.13 [73] using Flye version 2.9 [74] and racon version 2.24 [75], yielding a single 2.03 Mb contig.

The assembly of *S. pneumoniae* R6 *cps*_99-4038_ was aligned to the genome of *S. pneumoniae* R6 using lastz version 1.04.15 [76]. This alignment was filtered to single-fold coverage of the R6 genome with single_cov2 version 11 from the multiz package [77]. The resulting multiple alignment format output was used to generate a FASTA alignment with maf2fasta version 3 [77]. Recombinations were then identified using the pairwise mode of Gubbins v3.2.1 [40].

### Microscopy of pneumococci

Light microscopy of colonies used a Leica DFC3000 G microscope. To prepare samples for transmission electron microscopy, single pneumococcal colonies were vitrified using a Bal-Tec HPM 010 high pressure freezer and then transferred to a Leica EM AFS2 freeze substitution device. The samples were then substituted with 0.1% tannic acid and 0.5% glutaraldehyde in acetone at −90 °C for 72 h, washed three times in cold acetone over 2 h, then infiltrated with 2% osmium tetroxide and 1% uranyl acetate in acetone at −80 °C for 2 h. The temperature was then raised to −20 °C over 16 h, and then raised to 4 °C over 4 h. The samples were rinsed three times in acetone at room temperature and infiltrated with increasing concentrations of Epon before polymerisation at 60 °C for 48 h. Ultrathin 50-nm sections were cut on a Leica UC6 ultramicrotome using a diamond knife, mounted on uncoated copper grids, and further contrasted with uranyl acetate and lead citrate. Images were recorded on an FEI 120 keV Biotwin transmission electron microscope with a Tietz F4.16 Charge-Coupled Device.

## Results

### GPSC12 can be divided into distinct clades

**Fig. 1:**
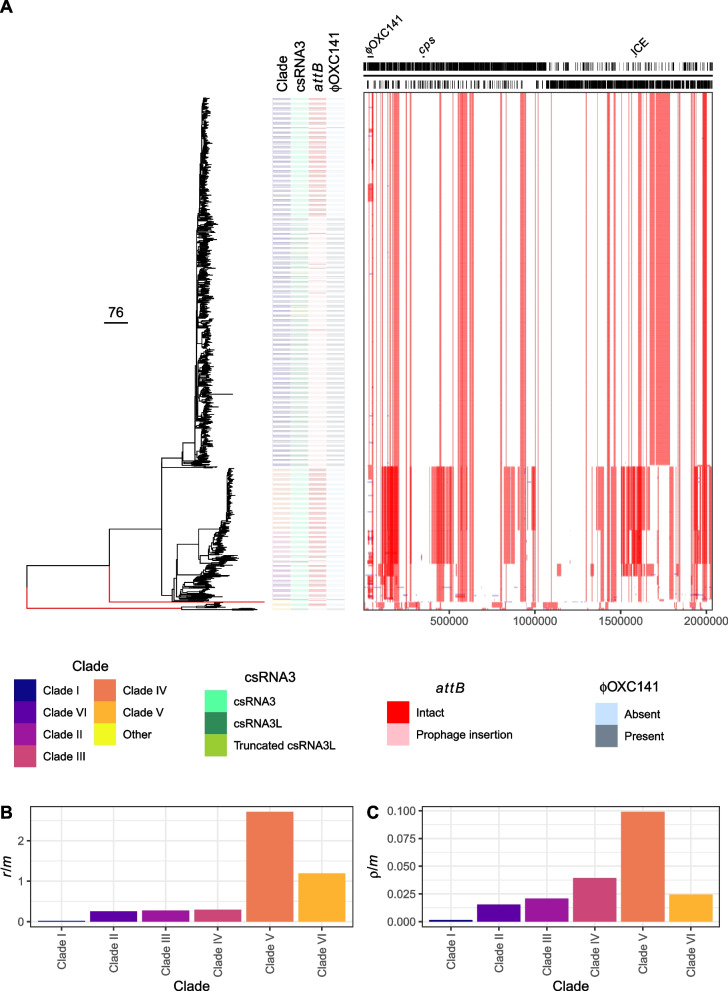
Epidemiology of *S. pneumoniae* GPSC12. **A** Evolutionary reconstruction of GPSC12. The left panel shows a recombination-corrected maximum-likelihood phylogeny of GPSC12, with branch lengths in point mutations per genome. The branches coloured red have been truncated to a length of 250 point mutations; the unmodified tree is shown in Additional file 1: Fig. S2, and can be interactively visualised using Microreact (https://microreact.org/project/gpsc12-recombination-dynamics). The adjacent columns describe the distribution of genetic characteristics across the collection, with one row per isolate, according to the legend at the bottom of the panel. The leftmost column assigns isolates to clades; the next column displays whether the unmodified csRNA3 or phage-modified csRNA3L was detected in the isolate; the next column shows whether *attB*_OXC_ is intact or disrupted by a prophage insertion; and the rightmost column shows whether a ϕOXC141-like prophage is present in the isolate. The right panel shows the distribution of inferred recombination events across the strain using a grid in which each row corresponds to an isolate, and each column a base in the reference genome, the annotation of which is displayed across the top. The recombination events are coloured red if they were reconstructed as occurring on an internal branch, and therefore are shared by multiple isolates through common descent, or blue if they were reconstructed on a terminal branch, and are therefore unique to a single isolate. **B, C** Recombination dynamics within GPSC12. The extent of recombination inferred to occur within each clade is quantified as the ratio of base substitutions introduced by recombination, relative to the number occurring through point mutation (*r*/*m*), in **B**, and as the number of recombination events relative to point mutations (ρ/*m*) in **C**

Previous genomic analyses of serotype 3 isolates were synthesised to generate a dataset of 978 isolates, of which 971 were of sufficiently high quality to be assigned to GPSCs (Additional file 1: Fig. S1). This identified 891 GPSC12 isolates (Additional file 1: Fig. S2) from 24 countries (Additional file 1: Fig. S3), with the majority (73.3%) from the UK or USA. In agreement with previous analyses of GPSC12, the recombination-corrected GPSC12 phylogeny demonstrated the strain was divided into multiple distinct clusters of isolates (Fig. 1, https://microreact.org/project/gpsc12-recombination-dynamics) [29–31]. In the original recombination-corrected phylogeny of GPSC12, isolates were divided into Clades I, II and III [29]. In an expanded dataset, Clade II was found to form a paraphyletic cluster subtending Clade I in a tree calculated from an alignment of core genome polymorphisms. Consequently, these three clades were renamed Clades I-ɑ, I-β and II, respectively [30]. However, this reclassification appears to be contingent upon using maximum likelihood phylogenies that were not corrected for recombination [30, 31], as trees accounting for sequence exchanges did not support the renaming [29, 36]. Accordingly, neither a neighbour-joining analysis of raw genetic distances, nor a recombination-corrected maximum likelihood phylogeny, supported Clades I-ɑ and I-β sharing a common ancestor that is exclusive of other isolates in this dataset (Fig. 1A, Additional file 1: Fig. S2). Hence, the initial classification into Clades I-III was expanded to six clades (Clades I-VI; Additional file 2: Table S1), enabling the detailed description of the greater diversity within GPSC12 revealed by recent genomic surveillance of the strain [31].

**Fig. 2:**
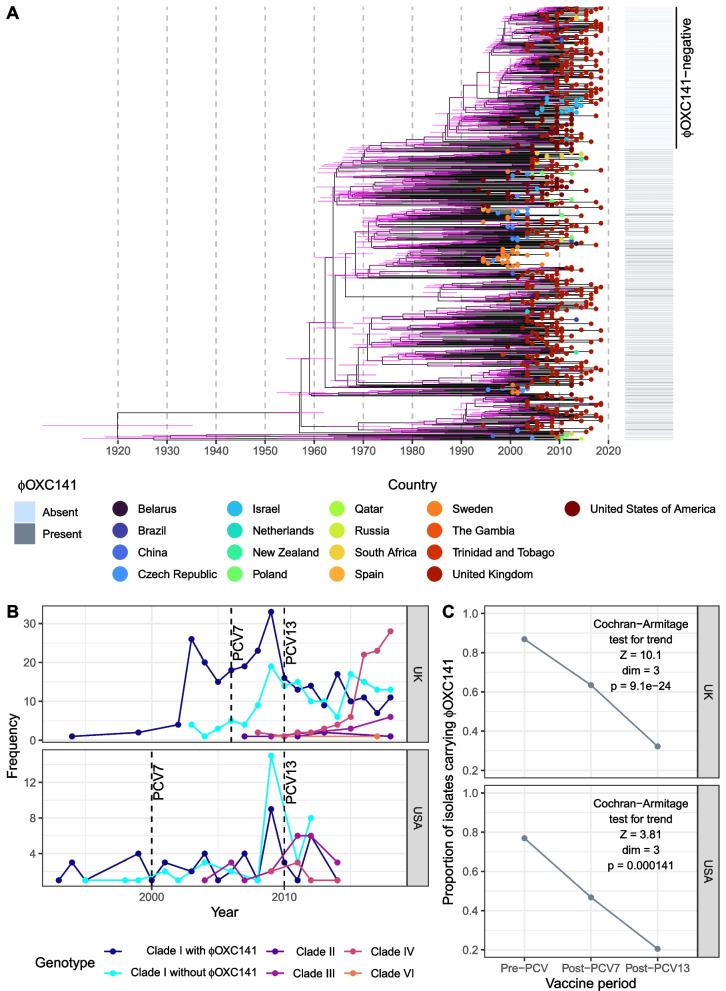
Population dynamics of GPSC12. **A** Time-calibrated phylogeny of GPSC12 Clade I. The horizontal axis relates the tree to dates. The magenta horizontal bars show the 95% credibility intervals for the date of each node in the phylogeny. The distribution of the ϕOXC141 prophage is shown by the column adjacent to the tree. The largest ϕOXC141-negative clade is annotated. **B** Temporal trends in the frequency of genotypes in the UK and USA, divided by assignment to clade. Clade I isolates are further subdivided by whether or not they carried a ϕOXC141-like prophage. The vertical dashed lines show the years in which PCVs were introduced in the UK and USA. **C** Line plot showing the decline in the prevalence of ϕOXC141-positive genotypes in the UK and USA GPSC12 populations following the introduction of PCVs

The majority (71.9%) of isolates belonged to Clade I (Additional file 1: Fig. S4), which comprised representatives from every inhabited continent, and included the reference genome, *S. pneumoniae* OXC141 [29]. Phylodynamic analyses estimated this clade originated around 1919 (95% credible interval: 1903–1934; Fig. 2A, Additional file 1: Fig. S5). Most Clade I isolates retained the ϕOXC141 prophage (Fig. 1A, Additional file 1: Fig. S6) integrated at the *attB*_OXC_ insertion site (Fig. 1A, Additional file 1: Fig. S7) [32], despite experimental evidence of phage targeting this site also integrating at an alternative secondary location [78]. However, in addition to the known sporadic loss of the prophage [29, 30], a 210-isolate ϕOXC141-negative subclade was revealed within Clade I (32.8% of the clade; Figs. 1A and 2A). Yet there were apparent cases of ϕOXC141 reacquisition within this clade, and the presence of ϕOXC141-like prophage in other clades suggested occasional within-strain transmission of this virus, as similar elements are very rare outside of GPSC12 (Additional file 1: Fig. S8). Examples of ϕOXC141-like prophage were even identified in the distantly related Clade V, detected in South-East Asia, containing isolates that have diverged from Clade I over centuries [36]. The other clades defined using the tree were more closely related to Clade I. The previously defined Clade II was split into Clades II and VI, the latter of which was enriched for isolates from China and South America. Similarly, within the globally disseminated Clade III, Clade IV was identified as mainly consisting of isolates from the UK (Fig. 1A). Hence, the ϕOXC141 prophage has infrequently transmitted to genotypes outside of Clade I, but its prevalence largely reflects the stable vertical inheritance of the insertion that is likely to have been present in the most recent common ancestor of Clade I.

### Ongoing diversification of GPSC12 in the UK and USA

The systematically collected UK dataset [31] allowed the pre- and post-PCV13 clade prevalences to be analysed and compared with the smaller longitudinally collected dataset from the USA (Fig. 2B). In the UK, Clade IV rose gradually in frequency following the introduction of PCV13 in 2010, then jumped in prevalence 3.7-fold to have a frequency similar to that of Clade I in 2015. The prevalence remained elevated in subsequent years, consistent with the patterns reported previously [31].

Yet changes within Clade I were evident in the UK pre-PCV13. The largest rise in the frequency of ϕOXC141-negative Clade I isolates occurred shortly after the introduction of PCV7 in the UK.  The ϕOXC141-positive and ϕOXC141-negative Clade I isolates were approximately equally common by the time PCV13 was introduced in 2010. These changes were mainly driven by the expansion of the ϕOXC141-negative subclade (Fig. 2A). Phylodynamic analysis suggested this clade originated around 1973 (95% credible interval: 1969 to 1977), but most isolates share a more recent common ancestor that existed in the 1990s (Fig. 2A). Both ϕOXC141-positive and ϕOXC141-negative Clade I isolates subsequently followed similar trends in the post-PCV13 era, with evidence of similar patterns in the USA (Fig. 2B). Hence, the post-PCV7 changes within Clade I and post-PCV13 expansion of other clades both contributed to the significant decline in ϕOXC141-positive genotypes in the UK and USA (Fig. 2C).

The reconstruction of the strain’s diversification also suggested variation in the recombination dynamics across GPSC12. To quantify divergence through homologous recombination only, statistics were calculated excluding recombinations affecting ϕOXC141 and a putative ICE within the reference genome of OXC141 [29, 33]. By both the *r*/*m* (ratio of base substitutions introduced by homologous recombination to those resulting from point mutation) and *ρ*/*m* (ratio of recombination events to point mutations) measures, Clade I underwent substantially less recombination than the other GPSC12 clades (Fig. 1B, C, Additional file 3: Table S2), consistent with previous analyses [30, 33]. The large number of Clade I isolates in the dataset make it unlikely the absence of detected recombinations reflects insufficient sampling of these genotypes. One parsimonious explanation for the lack of recombination in Clade I, and the rise in non-Clade I GPSC12 genotypes, is that Clade I produces a more extensive serotype 3 capsule. This could both limit DNA uptake by the competence system and potentially render these isolates more susceptible to PCV13-induced anticapsular antibodies, explaining their post-PCV13 fall in prevalence.

### The type 3 capsule reduces variation through transformation

**Fig. 3:**
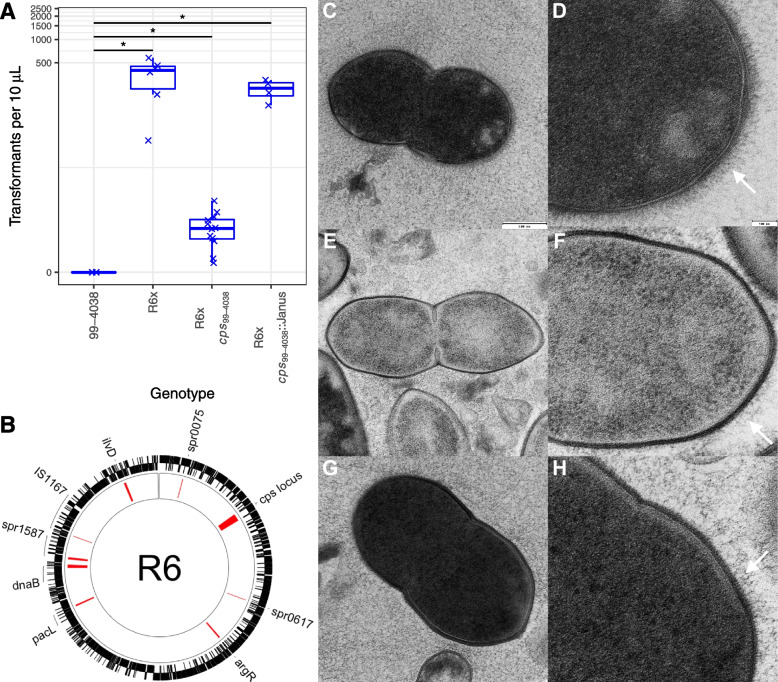
Reduction in transformation efficiency resulting from expression of the serotype 3 capsule. **A** Transformation efficiency of the Clade I isolate *S. pneumoniae* 99-4038; the unencapsulated laboratory isolate R6; the mutant R6 *cps*_99-4038_ (which carries the *cps* locus of *S. pneumoniae* 99-4038); the mutant R6 *cps*_99-4038_::Janus (in which the *cps* locus has been replaced by a resistance marker), and the mutant 99-4038 ΔϕOXC141 (in which the prophage was lost after exposure to mitomycin C). Each point represents an independent experiment, with the overall medians and interquartile ranges summarised by the box plots. A two-tailed Wilcoxon rank-sum test was used to compare the transformation frequencies of the genotypes to that of 99-4038, using a Holm-Bonferroni correction for multiple testing. Significance is coded as: *p* < 0.05, *; *p* < 0.01, **; *p* < 10^−3^, ***; *p* < 10^−4^, ****. **B** Recombinations inferred within R6 *cps*_99-4038_. The annotation of the recipient genome *S. pneumoniae* R6 is shown as a ring. The red blocks in the inner ring show the positions of inferred recombinations. Genes overlapping with these events are annotated around the edge of the panel. **C–H** Transmission electron microscopy showing the morphology of *S. pneumoniae* 99-4038, R6, and R6 *cps*_99-4038_. The panels show *S. pneumoniae* 99-4038 **C** whole cell and **D** cell surface morphology; *S. pneumoniae* R6 **E** whole cell and **F** cell surface morphology; and *S. pneumoniae* R6 *cps*_99-4038_**G** whole cell and **H** cell surface morphology. The whole cell morphologies are shown at a consistent scale, relative to the indicated 500 nm bar. The cell surface morphologies are each shown relative to the 100 nm bar, with white arrows pointing to the layer likely representing wall teichoic acid

When the previously characterised Clade I representative *S. pneumoniae* 99-4038 [29] was transformed with a rifampicin resistance marker, very few colonies were recovered across multiple experiments (Fig. 3A). To ascertain whether this inability to recombine could be ascribed to the capsule, the *cps* locus was introduced into a version of the highly transformable unencapsulated laboratory isolate *S. pneumoniae* R6, modified by the removal of its phase-variable *ivr* restriction-modification locus [7, 59]. This donor and recipient pair mirrored that used in the original experiments that demonstrated DNA was the transforming material [79].

One transformant, *S. pneumoniae* R6 *cps*_99-4038_, was isolated and characterised through whole genome sequencing. Analysis of the recombinant identified nine transformation events in the R6 *cps*_99-4038_ genome (Fig. 3B). The largest of these was a 27,760-bp recombination that spanned the entire *cps* locus, importing the intact serotype 3 allele to replace the defunct serotype 2 locus of the recombination recipient (Additional file 1: Fig. S9).

The expression of the serotype 3 capsule was demonstrated using both light (Additional file 1: Fig. S10) and transmission electron microscopy (Fig. 3C–H). The electron micrographs revealed *S. pneumoniae* R6’s surface featured both a darkly stained cell wall and a layer of fine threads extending tens of nanometres further out, likely corresponding to wall teichoic acid (Fig. 3F) [80]. These structures were also evident in 99-4038 and R6 *cps*_99-4038_, which were surrounded by an additional diffuse, yet extensive, matrix of capsule polysaccharides that filled the intercellular spaces (Fig. 3D, H). Hence, R6 *cps*_99-4038_ expressed a thick capsule that replicated that of a natural serotype 3 isolate.

Transformation assays with R6, R6 *cps*_99-4038_, and a mutant in which the *cps* locus had been eliminated (*cps*_99-4038_::Janus) found expression of the capsule reduced transformation efficiency by ~100-fold (Fig. 3A). Yet R6 *cps*_99-4038_ was still detectably transformable. Hence, the type 3 capsule may contribute to the low *r*/*m* of GPSC12 overall, but is not a sufficient explanation for the absence of transformation observed in Clade I isolates in vitro or in epidemiological analyses. Given the precedent of mobile elements inhibiting pneumococcal transformation, the ϕOXC141 prophage was the next locus considered as a candidate for explaining Clade I’s genetic homogeneity.

### Variation in csRNA3 is commonly driven by prophage insertion at *attB*_OXC_

**Fig. 4:**
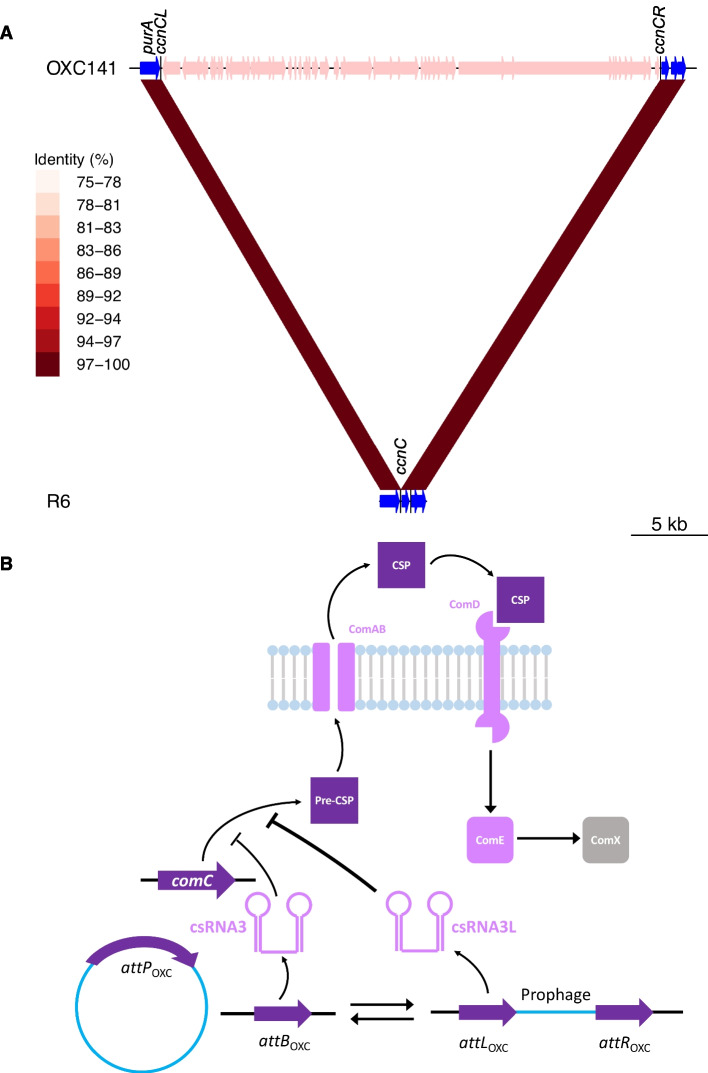
Modification of the csRNA3 sequence by prophage insertion. **A** Modification of *ccnC* through insertion of ϕOXC141 into *attB*_OXC_. The insertion of ϕOXC141, indicated by the pink genes integrated downstream of *purA*, into the *S. pneumoniae* OXC141 genome is compared to the unmodified *attB*_OXC_ site of R6 using BLASTN. The red bands link regions of similar sequence, with the colour indicating the level of sequence identity between the pair. The black vertical lines show how the cellular *ccnC* gene at the *attB* site is split into the *ccnCL* and *ccnCR* genes at the *attL* and *attR* sites, respectively. **B** Model of csRNA3 and csRNA3L-mediated inhibition of competence. Pneumococcal competence is regulated by a quorum-sensing mechanism. The *comC* gene encodes a pre-peptide that is exported and processed into competence-stimulating peptide (CSP) by the ComAB transporter. CSP is detected by the ComDE two competent system, which activates the transcription of early competence genes such as *comX*. The ComX alternative sigma factor then drives expression of the late competence genes required for uptake of DNA from the environment. The csRNA3 RNA inhibits the production of CSP by either blocking translation of the *comC* gene, or triggering degradation of the *comC* transcript. By reducing the autoinduction of the *comCDE* operon, the csRNA molecule suppresses further *comC* transcription. This delays or prevents the induction of competence by endogenously produced CSP, but does not affect the induction of competence by exogenously supplied CSP. This inhibition is affected by the interconversion between csRNA3P (encoded by *ccnCP* at *attP*) and csRNA3 (encoded by *ccnC* at *attB*), when a phage is excised from the chromosome, and csRNA3L (encoded by *ccnCL* at *attL*) and csRNA3R (encoded by *ccnCR* at *attR*), when the prophage is integrated. The enhanced activity of csRNA3L, represented by the thicker line indicating inhibition of *comC* expression, should therefore further delay or inhibit the spontaneous induction of the competence machinery, without affecting cell responses to CSP added in vitro

The *attB*_OXC_ site at which ϕOXC141 inserts was originally identified as intergenic [32], but has subsequently been shown to be within the *ccnC* gene, which generates the antisense non-coding RNA csRNA3 [78]. This is one of the *ccnA*-*E* genes in *S. pneumoniae*, encoding csRNA1-5, all of which target the *comC* gene [60], likely by either blocking its translation [60], or triggering degradation of the *comC* transcript through forming a double-stranded RNA complex [81]. The *comC* gene encodes the protein that is processed to generate competence-stimulating peptide (CSP), an autoinducing quorum-sensing pheromone necessary for pneumococci to undergo efficient transformation [82]. By limiting the production of CSP, csRNAs can delay, or even block, the positive feedback loop that results in the induction of competence for transformation [60, 83, 84]. Normally, insertion into the *ccnC* gene would be expected to cause a loss-of-function mutation, although the redundancy between csRNA genes would mean the phenotypic impact would likely be negligible [84]. However, the integration of ϕOXC141 instead generated two modified *ccnC*-like sequences: *ccnCL* at the *attL* site (encoding csRNA3L), and *ccnCR* at the *attR* site (encoding csRNA3R; Fig. 4A), as observed for phage ϕSpSL1 [78].

The *ccnCL* gene is expected to be functional, as its promoter is that of the native *ccnC* gene, and the 5′ stem-loop of csRNA3L is unmodified. However, the 3′ stem-loop is changed from having four unpaired bases in the loop, to instead comprising the sequence CAAUCA (Additional file 1: Fig. S11). This matches the sequences of the 3′ loops in csRNA1, csRNA3 and csRNA4, each of which is more effective at inhibiting competence than the unmodified csRNA3 allele [83]. This does not change the predicted affinity of the csRNA3 for the *comC* transcript (Additional file 1: Fig. S11), but may alter its interaction with Cbf1, which seems to stabilise and process these RNAs, or another of the regulatory or effector proteins with which these RNAs interact [85]. Hence, ϕOXC141 insertion has the potential to increase the effectiveness of a cell’s csRNA repertoire at preventing competence induction, thereby accounting for the reduced rate of homologous recombination observed in Clade I (Fig. 4B).

**Fig. 5:**
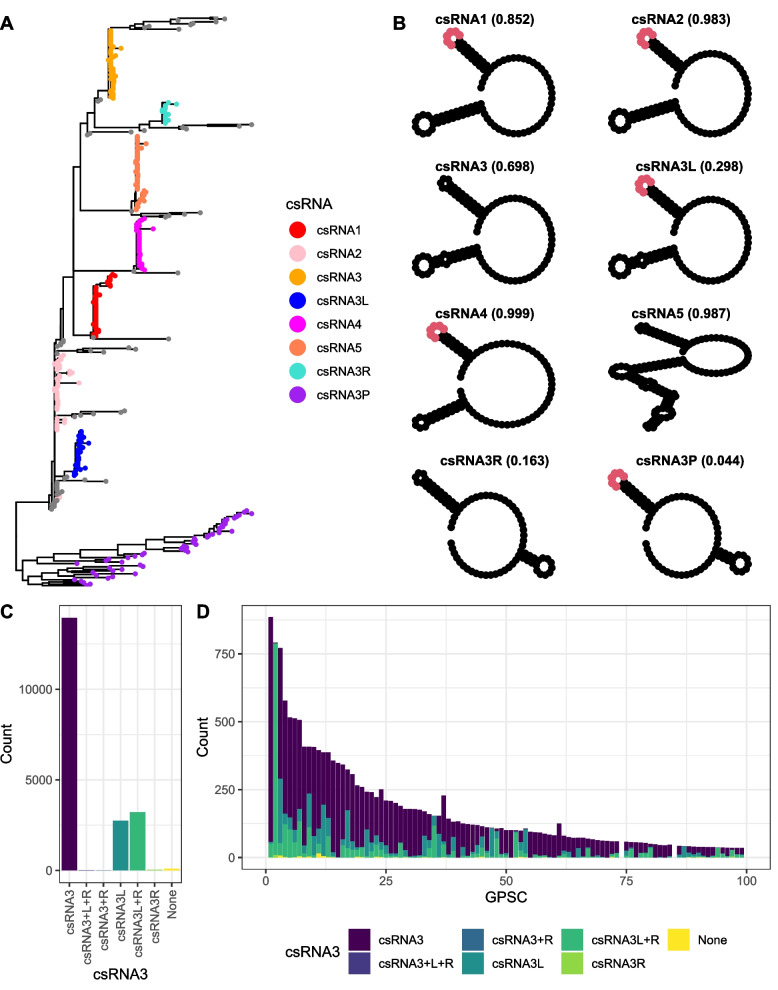
Distribution of csRNA sequences across the 20,047 Global Pneumococcal Sequencing (GPS) project isolates. **A** A maximum-likelihood phylogeny of 388 csRNA sequences, representing the non-redundant set identified within the GPS collection after excluding examples that were <80 nt in length or contained ambiguous bases. Nodes are coloured according to assignment to the eight different csRNA types shown in panel (**B**). **B** The predicted structure of the most common sequence of each csRNA type is shown. The sequence CAAUCA is highlighted in red where it is present. The total frequency of all sequences assigned to the named allele in the GPS isolates is shown in parentheses. **C** The distribution of csRNA3 allele combinations across the GPS collection. The bar chart quantifies the prevalences of different combinations of the unmodified csRNA3 type, and the prophage-modified csRNA3L and csRNA3R types, across isolates. **D** Distribution of different csRNA3 type combinations across Global Pneumococcal Sequence Clusters 1 to 99 in the GPS collection

To ascertain whether the modification of csRNA was common across the species, the 20,047 pneumococcal genomes from the GPS project were scanned for *ccnA*-*E* genes [3]. This identified 104,934 csRNA sequences, with a mode of five per genome (Additional file 1: Fig. S12). A phylogeny of a non-redundant set of 388 sequences enabled the majority of csRNA alleles to be classified into eight sets (Fig. 5A; Additional file 4: Table S3). Three of the csRNAs (csRNA2, csRNA4 and csRNA5) were present in >98% of isolates, exhibiting very little variation across the species (Fig. 5B). The csRNA1-type sequences were only present in ~85% of isolates. The corresponding *ccnA* gene is found in tandem with *ccnB* (encoding csRNA2) upstream of *ruvB* [83]. An independent analysis of this chromosomal locus found it was structurally divergent from the version in *S. pneumoniae* R6 in ~18% of isolates (Additional file 1: Fig. S13). Within Clade I, a comparison of the complete genomes of *S. pneumoniae* OXC141 and 99-4038 suggested this was the result of an intragenomic recombination between *ccnA* and *ccnB* (Additional file 1: Fig. S13). The chimeric *ccnAB* sequences produced by these events were classified as csRNA2, as they retained the 5′ stem-loop of csRNA1, which is similar between both genes, and the 3′ stem-loop of csRNA2, which is divergent from that of csRNA1 (Fig. 5B).

The csRNA3 and csRNA3L sequences were present in 69.8 and 29.8% of isolates respectively, but never found within the same isolate (Fig. 5C). This indicates almost all isolates had one of these two mutually exclusive alleles. Over half of isolates with a csRNA3L were also found to have a csRNA3R (present in 16.3% of isolates), which was almost never found in the absence of csRNA3L. The inability to detect csRNA3R in many csRNA3L-positive genomes may represent the reduced functional constraints on csRNA3R sequences, as their predicted structure features a short stem-loop that suggests they may not form an effective csRNA (Fig. 5B). The same small stem-loop is present on a representative of a diverse set of sequences labelled csRNA3P, which are most divergent from the previously characterised csRNA sequences (Fig. 5B). The corresponding *ccnCP* genes can be found at the *attP* sites of phage genomes, between the integrase and lytic amidase genes (Additional file 1: Fig. S14) [78]. Correspondingly, the csRNA3P stem-loop that does not match with csRNA3R is that which replaces the 3′ stem-loop of csRNA3 to generate csRNA3L (Fig. 5B). Hence, variable *ccnCP* sequences carried on diverse prophage modify the cellular *ccnC* gene through insertion to generate *ccnCL* and *ccnCR* genes (Fig. 4B). The widespread alteration affects approximately one-third of pneumococci.

### Modification of csRNA3 in serotype 1 and genetically tractable genotypes

To test whether the integration of ϕOXC141 inhibited the transformation of Clade I, *S. pneumoniae* 99-4038 was passaged in the presence of the lysogen inducer mitomycin C [32], enabling the isolation of bacteria lacking the prophage (ΔϕOXC141; Additional file 1: Fig. S15). When competence was induced by exogenous CSP (Fig. 4B), the transformation of ΔϕOXC141 was as inefficient as transformation of the parental genotype (Fig. 3A). This suggested 99-4038 would be a poor model for understanding the effects of csRNA3 alteration, as this change was hypothesised to inhibit endogenous CSP production, the quantification of which requires lower-efficiency assays of spontaneous transformation. Nevertheless, the frequency of *ccnCL* in the pneumococcal population made it possible to search for a genetically tractable isolate carrying this prophage-modified sequence.

The *ccnCL*-type sequences were detected in almost half of GPSCs (292 of 586; 49.8%), and were almost ubiquitous within GPSC2, the most common strain expressing serotype 1 (Fig. 5D) [3]. This strain is responsible for a high proportion of invasive disease in Africa and is regarded as being unusually genetically homogenous [3, 86]. The modification of csRNA3 results from the insertion of ϕPNI0373 at *attB*_OXC_ (Additional file 1: Fig. S16) [87]. Analysis of the GPS collection suggests this prophage is conserved between almost all GPSC2 isolates, despite being absent from all other GPSCs (Additional file 1: Fig. S17). Yet generating mutant variants of serotype 1 pneumococci is challenging [87]. Instead, the effects of csRNA3L were investigated in GPSC97 isolate *S. pneumoniae* RMV8, in which competence for transformation can be efficiently induced by exogenous CSP [63, 64]. Although this isolate has a CSP2 pherotype, rather than the CSP1 pherotype of Clade I, the interactions of both csRNA3 and csRNA3L with the *comC* transcripts generating these CSP types were predicted to be similar (Additional file 1: Fig. S11).

The modification of *ccnC* in *S. pneumoniae* RMV8 resulted from the integration of a 32.8-kb prophage, ϕRMV8, at *attB*_OXC_ (Additional file 1: Fig. S18). Previous experimental work on this genotype resulted in the isolation of two variants (Additional file 1: Fig. S19), each expressing different alleles of the phase-variable SpnIV restriction-modification system [64], encoded by the translocating variable restriction (*tvr*) locus [4]. Further alterations at the *tvr* loci of these isolates were precluded by the disruption of the *tvrR* gene, encoding the recombinase responsible for rearrangements through excision and integration (Additional file 1: Fig. S19) [64]. The more common “dominant” variant (RMV8_domi_) expressed a SpnIV allele that methylated the motif GATAN_6_RTC, whereas the allele expressed by the less common “rare” variant (RMV8_rare_) methylated the motif GTAYN_6_TGA. Comparing the growth curves of these variants, and *tvr*::*cat* derivatives in which the *tvr* locus was replaced with a chloramphenicol resistance marker, had found RMV8_rare_ exhibited a growth defect in late exponential phase (Additional file 1: Fig. S20). This correlated with increased activity of ϕRMV8 in RMV8_rare_ during stationary phase, and correspondingly, the growth defect was not observed when the prophage was removed in an RMV8_rare_ ϕRMV8::*tetM* mutant (Additional file 1: Fig. S20). Hence, the distinctive growth profile of RMV8_rare_ was the consequence of increased activation of ϕRMV8. This correlated with different patterns of epigenetic modifications of the pneumococcal chromosome, analogous to the variation in PRCI activity seen between *tvr* variants in another pneumococcal genotype [63].

### Prophage-driven alteration of csRNA3 affects *comC* expression

**Fig. 6:**
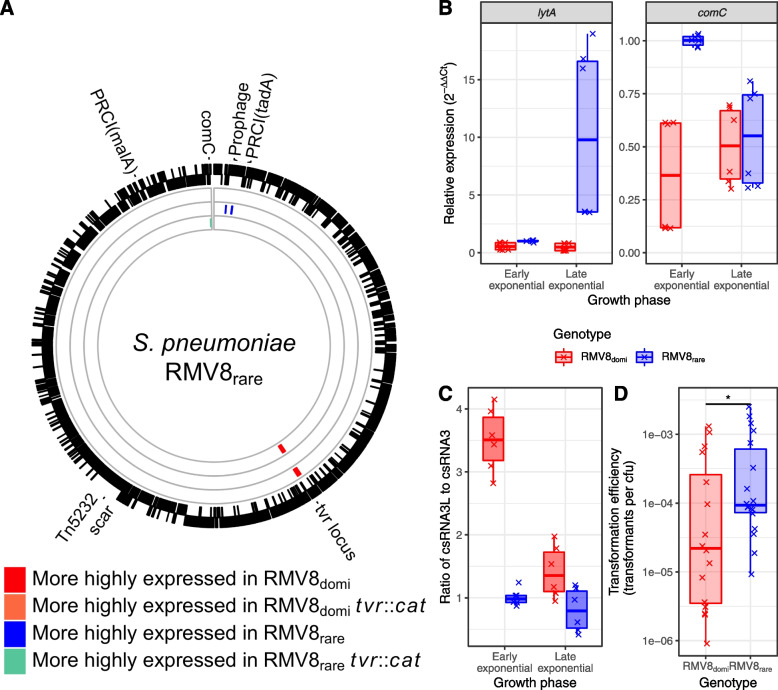
Variation between RMV8 genotypes. (A) RNA-seq analysis of *S. pneumoniae* RMV8. The outer rings represent the annotation of the complete genome of the *S. pneumoniae* RMV8_rare_ variant (accession code OX244288). Labels mark the positions of the mobile elements present in this isolate, corresponding to a prophage, two phage-related chromosomal isolates (PRCIs; inserted near *tadA* and *malA* [4]) and a Tn*5253* scar [88]. The inner rings show the differences in expression between RMV8_domi_, the most frequently isolated genotype in RMV8 cultures, and derived genotypes. The outermost of these rings shows the comparison with RMV8_domi_*tvr*::*cat*, in which the *tvr* locus was replaced with a *cat* resistance marker. The next ring shows the comparison with RMV8_rare_, in which significant differences at the prophage locus were detected. The innermost ring shows the comparison with RMV8_rare_*tvr*::*cat*, which highly expressed the *comC* gene. **B** Quantification of ϕRMV8 *lytA* and *comC* expression by qRT-PCR in RMV8_domi_ and RMV8_rare_ samples collected at early (OD_600_ = 0.2) and late (OD_600_ = 0.5) exponential phase growth. Six points are shown, corresponding to three technical replicate measurements of each of two biological replicates. Data are coloured according to the genotype being tested. **C** Quantification of the relative levels of csRNA3L and csRNA3 in early and late exponential phase growth. Data are shown as in panel (**B**). **D** Scatterplot showing the spontaneous transformation efficiency of the RMV8 variants. Each point represents an independent experiment, with the overall medians and interquartile ranges summarised by the box plots. A two-tailed Wilcoxon rank-sum test was used to compare the transformation frequencies of the variants. Significance is coded as: *p* < 0.05, *; *p* < 0.01, **; *p* < 10^−3^, ***; *p* < 10^−4^, ****

To understand how differences in ϕRMV8 activity might affect csRNA3-type sequences, RNA-seq data were generated for three replicates of each of the four RMV8 genotypes (the two variants, and the corresponding *tvr*::*cat* mutants) during the late exponential growth phase (Additional file 7: Table S6). The sequence reads exhibited consistent inferred fragment size (Additional file 1: Fig. S21) and gene expression (Additional file 1: Fig. S22) distributions, suggesting the datasets should be informatively comparable. Comparing RMV8_domi_ with the other three genotypes using Q-Q (Additional file 1: Fig. S23) and volcano (Additional file 1: Fig. S24) plots suggested the main differences between the genotypes could be captured with a false discovery rate threshold of 10^−3^ (Additional file 8: Table S7). As a positive control, this found the *tvr* loci to be more highly expressed in RMV8_domi_ than either of the genotypes in which these genes were deleted (Fig. 6A).

The only two other loci to exhibit significant differences in transcription were ϕRMV8 and *comC* (Additional file 9: Table S8). Consistent with the growth inhibition attributable to elevated ϕRMV8 activity in RMV8_rare_, the prophage was more highly transcribed in this genotype across the lysogeny (Additional file 1: Fig. S25), replication (Additional file 1: Fig. S26), structural (Additional file 1: Fig. S27) and lysis (Additional file 1: Fig. S28) genes, although not all reached statistical significance (Fig. 6A). This heightened prophage activity was not evident in RMV8_rare_*tvr*::*cat*, which instead upregulated *comC* relative to the other genotypes (Fig. 6A). The co-operonic *comDE* genes, encoding the CSP receptor, were also more highly transcribed in this genotype (Additional file 1: Fig. S29). Hence, the RNA-seq data demonstrated a specific link between a prophage at *attB*_OXC_ and transcription of the *comCDE* operon.

As conventional RNA-seq library preparation does not efficiently incorporate small non-coding RNAs, understanding whether csRNA3 connected the changes in *comCDE* and ϕRMV8 transcription required quantitative reverse transcriptase PCR (qRT-PCR) experiments. Samples were taken during early exponential growth (OD_600_ = 0.2), when competence is typically inducible, and late exponential growth (OD_500_ = 0.5), when the RNA-seq samples were collected. Comparisons of RMV8_domi_ and RMV8_rare_ confirmed the ϕRMV8 *lytA* gene was more active in the latter genotype (Fig. 6B). The increased excision of ϕRMV8 in RMV8_rare_ (Additional file 1: Fig. S20) raised the expression of csRNA3, from the restored *ccnC* gene, relative to csRNA3L (Fig. 6C). This ratio was highest during early exponential phase, and correspondingly *comC* expression in RMV8_rare_ was approximately twice that of RMV8_domi_ at this growth stage (Fig. 6B). This was sufficient to significantly alter the induction of competence, as RMV8_rare_ underwent spontaneous transformation fourfold more frequently than RMV8_domi_ (Fig. 6D), despite there being no significant difference in their transformability when CSP was added exogenously [63]. Hence, more stably integrated prophage correlated with a higher ratio of csRNA3L to csRNA3 expression, lower *comC* transcription and reduced transformability.

Consistent with the RNA-seq data, *comC* expression was similar in RMV8_domi_ and RMV8_rare_ in late exponential phase (Fig. 6B), but elevated in RMV8_rare_*tvr*::*cat* (Additional file 1: Fig. S30). This appeared to reflect the low levels of csRNA3 and csRNA3L in RMV8_rare_*tvr*::*cat* during early exponential phase (Additional file 1: Fig. S30), which correlated with the low activity of ϕRMV8 in this genotype (Additional file 1: Fig. S20 and S30). However, the measurement of csRNA3L levels and ϕRMV8 transcription by qRT-PCR were not independent. Mapping RNA-seq data to the *attL*_OXC_ site demonstrated antisense expression of *ccnCL* was driven by transcription initiated within ϕRMV8 while it was integrated in the chromosome (Additional file 1: Fig. S30). This has the potential to confound the quantification of csRNA3L levels in lysogenic genotypes, as the variable intracellular dynamics of prophage excision and reintegration changed the expression of csRNA3 and csRNA3L between even near-isogenic cells.

### Expression of csRNA3L inhibits activation of transformation through quorum sensing

**Fig. 7:**
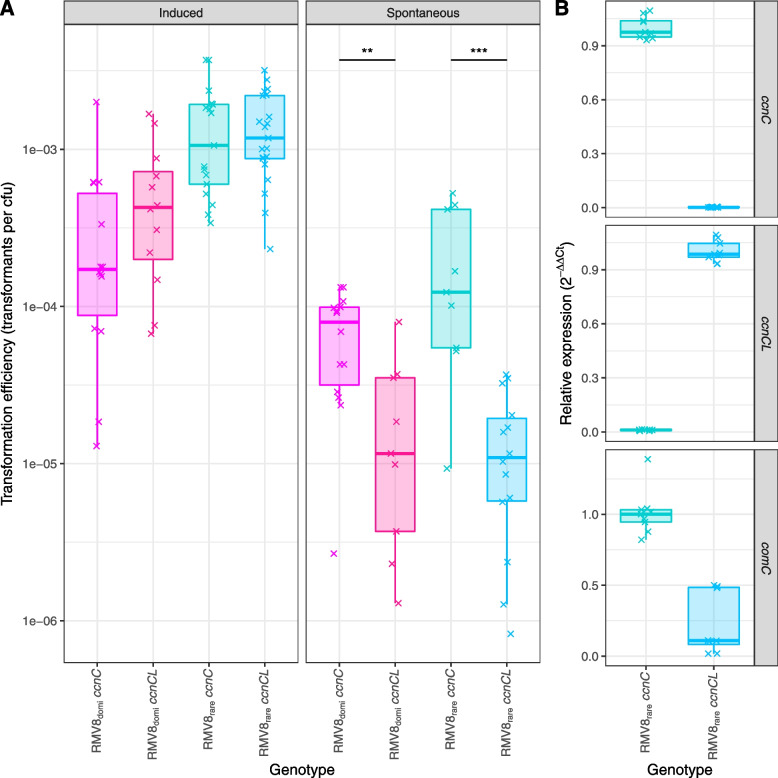
The increased inhibition of competence induction by *ccnCL* relative to *ccnC* in RMV8. **A** Transformation frequencies of independent RMV8_rare_ and RMV8_domi_ mutants expressing *ccnC* and *ccnCL*. Frequencies were calculated as the count of rifampicin-resistant colonies relative to the number of colony-forming units (cfu) in the sample. Each point represents an independent experiment, summarised for each genotype as a boxplot. Data are coloured according to genotype. Induced competence experiments measured the frequency of recombination following the addition of exogenous CSP. Spontaneous competence experiments measured the frequency of recombination in overnight cultures with no added CSP. A two-tailed Wilcoxon rank-sum test was used to compare the transformation frequencies of the matched *ccnC* and *ccnCL* mutants within each background, using a Holm-Bonferroni correction for multiple testing. Significance is coded as: *p* < 0.05, *; *p* < 0.01, **; *p* < 10^−3^, ***; *p* < 10^−4^, ****. **B** Levels of *ccnC*, *ccnCL* and *comC* transcripts in RMV8_rare_ mutants expressing either *ccnC* or *ccnCL* at the end of spontaneous transformation experiments. RNA concentrations were estimated using qRT-PCR and quantified using the ΔΔCt approach (see “Methods”). The points represent the data from three technical replicate measurements of each of three biological replicates

To establish whether csRNA3L caused the differences in transformation efficiency required the construction of mutants stably expressing one allele at the *attB*_OXC_ site. Both *ccnCL* and ϕRMV8 were replaced with a single fixed csRNA3-type sequence in RMV8_rare_ and RMV8_domi_: either *ccnC* from R6 or *ccnCL* from RMV8_rare_. No substantial growth difference was observed between the two genotypes in vitro (Additional file 1: Fig. S31). Spontaneous transformation assays demonstrated both *ccnC* mutants exhibited significantly higher transformation efficiencies than the corresponding *ccnCL* mutants: an 11-fold difference in RMV8_rare_, and an eight-fold difference in RMV8_domi_ (Fig. 7A). Negative control experiments, conducted in the absence of DNA or the presence of DNase I, confirmed these assays should accurately reflect levels of spontaneous transformation (Additional file 1: Fig. S31)

By contrast, when competence was induced with exogenous CSP during early exponential phase growth, no significant difference was observed between the mutants in the RMV8_rare_ or RMV8_domi_ backgrounds (Fig. 7A). Hence, the observed differences between the *ccnC* and *ccnCL* genotypes did not represent variation in the activity of the competence machinery itself. To test the hypothesised causal mechanism, the expression of csRNA3, csRNA3L and *comC* was quantified in the RMV8_rare_ mutants under the conditions used to assay spontaneous transformation (Fig. 7B). These confirmed csRNA3 and csRNA3L were only detectable in the corresponding mutants. They also demonstrated that *comC* expression was at least halved in the *ccnCL* mutant relative to the *ccnC* mutant. Therefore, prophage-mediated RNA alteration inhibited the ability of pneumococci to spontaneously induce competence through the endogenous production of a quorum-sensing signal.

## Discussion

Pneumococci have proved adept at acquiring antibiotic resistance and evading vaccine-induced immunity through transformation [89]. Yet GPSC12 isolates have persisted as a major cause of disease after the introduction of PCV13 while retaining their serotype 3 capsule and typically remaining pansusceptible to antibiotics [3]. This absence of adaptive evolution to public health interventions is the consequence of the strain undergoing relatively little diversification through homologous recombination, with the overall *r*/*m* of 0.56 more than an order of magnitude lower than that calculated from samples of commonly antibiotic-resistant strains such as GPSC1 [90], GPSC6 [35] or GPSC16 [33]. The mucoid serotype 3 capsule contributes to the low recombination rate, likely through acting as a physical barrier to the acquisition of exogenous DNA. Yet this is insufficient to explain the low frequency of recombinations in Clade I relative to the rest of GPSC12. The ϕOXC141 prophage is the main polymorphism distinguishing Clade I from other GPSC12 genotypes, and the demonstration that this element inhibits the induction of competence suggests it explains at least some of this difference. In the UK, systematic longitudinal sampling of GPSC12 demonstrated two separate processes both contributed to the primarily ϕOXC141-positive pre-PCV7 population being replaced by a largely ϕOXC141-negative post-PCV13 population.

Within Clade I, there was a reduction in the proportion of isolates carrying ϕOXC141 post-PCV7. This was driven by the expansion of a subclade lacking ϕOXC141, suggesting this element is deleterious to its host cell, albeit not causing such a large cost that would drive a rapid selective sweep of ϕOXC141-negative genotypes. The monophyletic expansion of the large ϕOXC141-negative clade indicates the prophage is infrequently lost [91], consistent with the rarity of ϕOXC141-negative isolates in mitomycin C passages (Additional file 1: Fig. S17).

After the introduction of PCV13, Clade I was replaced by clades with a greater propensity to acquire diverse sequence through recombination. There is no evidence that this can be directly ascribed to differential effects of vaccine-induced immunity, based on physiochemical and serological assays applied to different clades previously [30]. Yet given that Clade I is decades old [29, 30], and the composition of GPSC12 changed rapidly in the UK just as the prevalence of other strains was shifting following the introduction of PCV13 [92], it is likely that these changes reflect the indirect consequences of vaccine-induced immunity on the bacterial population [93].

This decline in the prevalence of ϕOXC141, and the associated csRNA3L modification, likely reflects the selection against prophage infection that is necessary to preserve the non-lysogenic nature of many pneumococci [4]. That such a secular trend may have been accelerated by the vaccine-induced disruption of the pneumococcal population complicates the interpretation of the impact of PCVs. This replacement of the “frozen” Clade I post-PCV is consistent with the post-vaccine elimination of similarly slowly evolving genotypes [34, 35]. Another such low-diversity strain that has been effectively targeted by PCVs is GPSC2 [3, 86], the predominant serotype 1 genotype. GPSC2 also carries the csRNA3L modification as a consequence of the stable integration of the ϕNPI0373 prophage in the *attB*_OXC_ site. The long-term association between both GPSC2 and GPSC12 Clade I and their corresponding prophage means the csRNA3L modification will have affected the diversification of these two genotypes over much of their evolutionary history. By contrast, most prophage infections are ephemeral [4, 8]. Hence, the same modification is unlikely to have a detectable effect on the historical recombination rate of other isolates carrying prophage that only recently inserted at *att*_OXC_.

In the “frozen” genotypes, there are likely to be other contributing mutations that also limit the transformation rate. In GPSC2, the lack of transformation has been partially attributed to disruptive mutations in competence genes [87]. Similarly, the lack of diversification through homologous recombination in *S. pneumoniae* 99-4038 cannot be entirely explained by the phage-mediated alteration of *ccnC*. While the serotype 3 capsule substantially limits the efficiency of DNA import, transformants were still detectable in an R6 mutant expressing this capsule following addition of exogenous CSP, in contrast to *S. pneumoniae* 99-4038. This implies there is a difference in transformation efficiency that is independent of both the capsule and the ability to generate endogenous CSP. Such a mutation may be shared across GPSC12 or private to Clade I. This likely reflects the complexity of the regulation of transformation [6, 63], and the multiplicity of mechanisms that can block homologous recombination, such as restriction-modification systems [64, 94]. The relative importance of these multiple inhibitory mechanisms may be quantified by the ϕOXC141-negative subclade within Clade I, which has a restored *ccnC* (Fig. 1A). There is little evidence yet of an increased transformation rate in this group, but this is unsurprising, given its recent expansion, limited geographic sampling, and the low *r*/*m* across GPSC12 (Figs. 1 and 2). Yet if its dissemination continues, an increase in the rate of recombination from that of the rest of Clade I, to a level more similar to the rest of GPSC12, would be expected if *ccnCL* is important in inhibiting transformation relative to other aspects of the Clade I genotype.

Nevertheless, the alteration of csRNA3 is likely to be hugely influential on pneumococcal evolution at a broader scale, owing to this polymorphism being found in almost half of GPSCs, and almost one-third of individual pneumococcal isolates. Perhaps the most persuasive evidence of its importance is the conservation of the phage sequences contributing to the *ccnCL* allele, in contrast with the otherwise immensely variable nature of pneumococcal prophage [4, 32, 95]. This is indicative of a strong selective benefit of the csRNA3 alteration to the virus. Although the modification cannot prevent competence being induced by CSP produced by cells with an unmodified csRNA3 within the same nasopharynx, it should nevertheless be effective, as most individuals carrying pneumococci are singly colonised by cells descended from one recent acquisition [96]. In cases of multiple colonisation, it is likely that different strains are physically segregated within the nasopharynx as a consequence of interstrain competition mediated by mechanisms such as bacteriocins [97, 98]. This limits cross-talk between co-colonising strains, which is also inhibited by variation in CSP pherotypes [4, 98]. Hence, it is unlikely cells with unmodified csRNA3 sequences will frequently induce competence in cells encoding csRNA3L. This rarity of close interstrain interactions is consistent with the low frequency of sequence exchange between divergent donor and recipient cells [33].

Yet the rapid dissemination of phage throughout the pneumococcal species suggests these elements are effective at interstrain transmission [4, 8]. Through limiting transformation within communities of cells, prophage generating *ccnCL* alleles can reduce the rate at which they are deleted as they spread between previously uninfected cell populations within a host nasopharynx [8, 99]. Notably, the *ccnCL* allele is only present when the prophage is integrated into the chromosome, and therefore vulnerable to deletion through “chromosomal curing”, with the original *ccnC* restored on phage excision (Fig. 4B). Hence, the alteration of the cell’s csRNA repertoire can be explained as a defence mechanism of a selfish MGE.

This is the second of the four commonly targeted pneumococcal *attB* sites [100] at which prophage integration has been found to inhibit transformation. Prophage insertions disrupting the pilus gene *comYC*, which is necessary for competence [34, 89], cause loss of function, contrasting with the increased effectiveness of the phage-modified competence-inhibiting *ccnCL*. The truncation of *comYC* was recently demonstrated to be the most common single type of protein-modifying mutation affecting coding sequences during within-host pneumococcal evolution [96]. This emphasises the importance of conflict between prophage and the host cell in rapidly generating common polymorphisms in this bacterium [99].

While the disruption of the competence machinery by mobile elements has already been shown to be widespread across bacterial pathogens [8], the potential for blocking transformation through modification of regulatory non-coding RNAs is becoming increasingly apparent. In addition to csRNAs, another pneumococcal non-coding RNA has been found to inhibit transformation by repressing *comD* expression [101]. In *Legionella pneumophila*, the RocR non-coding RNA was identified as a repressor of competence [102]. Subsequently, a set of *L. pneumophila* mobile elements were found to carry a modified RocRp RNA, enabling the elements to inhibit transformation through a mechanism analogous to the modification of csRNA3 [103]. Hence, the manipulation of competence regulatory systems as part of the conflict between horizontal DNA transfer mechanisms is likely to emerge as an important factor in shaping genetic diversity across more species as our understanding of mobile elements and transformation regulation improves. These trends must then be disentangled from the effects of vaccine and antibiotic selection to understand the epidemiology of bacterial pathogens.

## Conclusions

PCVs cause substantial changes in pneumococcal populations, resulting from both the direct consequences of vaccine-induced immunity, and indirect effects as the population is restructured. The changes in the GPSC12 population likely reflect the replacement of genotypes suffering the fitness cost of infection with a prophage. This burden was further exacerbated by the intragenomic conflict between mobile elements and the transformation machinery that resulted in the integrated phage stabilising its vertical inheritance through inhibiting the host cell signalling needed to induce competence. Hence, interpreting the genomic epidemiology of bacteria requires consideration of their biology and genetics in conjunction with immunological data.

## Supplementary Information

**Additional file 1: Figures S1-S31.** All supplementary figures included in this study.

**Additional file 2: Table S1.** Accession codes and epidemiological data for the 891 GPSC12 isolates analysed in Fig. 1.

**Additional file 3: Table S2.** Statistics on the diversification of Clades I-VI within GPSC12 calculated from the evolutionary reconstruction by Gubbins.

**Additional file 4: Table S3.** Distribution and sequences of the types of csRNAs identified in this work.

**Additional file 5: Table S4.** Genotypes used in experiments described in this study.

**Additional file 6: Table S5.** Oligonucleotides used in experiments described in this study.

**Additional file 7: Table S6.** Accession codes of the RNA-seq datasets.

**Additional file 8: Table S7.** Statistical analysis of gene expression from RNA-seq data. All genetic features are labelled with their locus tags in the annotation of RMV8_rare_ (accession code OX244288). Each row describes the output for an individual gene in a single RNA-seq dataset.

**Additional file 9: Table S8.** Significant changes in expression identified by RNA-seq analyses. All the listed genes differed in expression between genotypes with a *q* value exceeding the threshold value of 10^−3^.

## Data Availability

Accession codes for the genomic data used to analyse the epidemiology of GPSC12 are listed in Additional file 2: Table S1. Accession codes for the genomic data used in the experimental analyses are listed in Additional file 5: Table S4. Accession codes for the RNA-seq data are listed in Additional file 7: Table S6. Epidemiological data and phylogenetic analyses are available from https://microreact.org/project/gpsc12-recombination-dynamics.
