# Supplementary material for: Post-vaccine epidemiology of serotype 3 pneumococci identifies transformation inhibition through prophage-driven alteration of a non-coding RNA
Source: Genome Med. 2022 Dec 20;14:144. doi: 10.1186/s13073-022-01147-2 (PMC9764711; doi:10.1186/s13073-022-01147-2)
Supplement: Supplementary file 1 — Additional file 1: Figures S1-S31. All supplementary figures included in this study. [file 13073_2022_1147_MOESM1_ESM.pdf]

<u>Supplementary Figures</u>

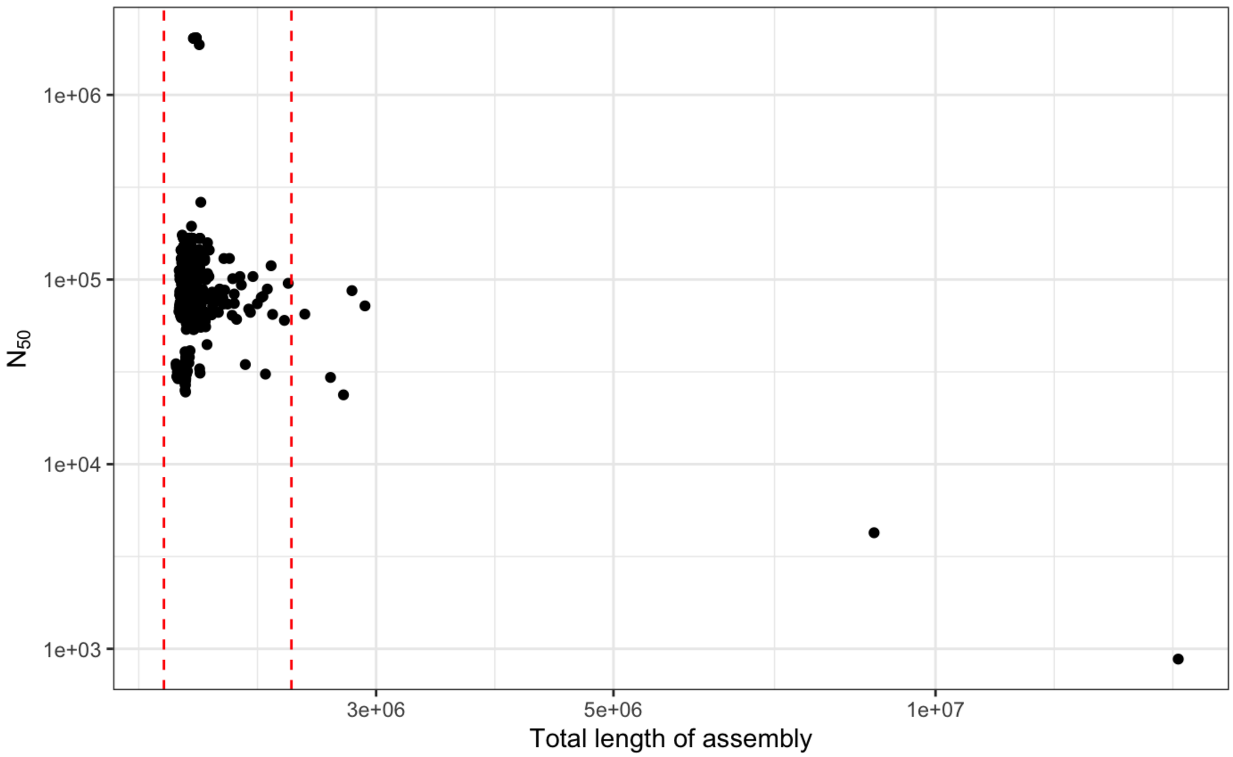

Figure S1 Exclusion of low-quality datasets. The scatterplot compares the length and N_{50} (the length of the contig spanning the midpoint of a draft assembly, when contigs are arranged in length order) of 978 assemblies generated with SPAdes. The vertical red dashed lines show the threshold assembly lengths of 1.9 Mb and 2.5 Mb that were used to identify anomalously long or short sequences. This resulted in the exclusion of seven assemblies from the dataset.

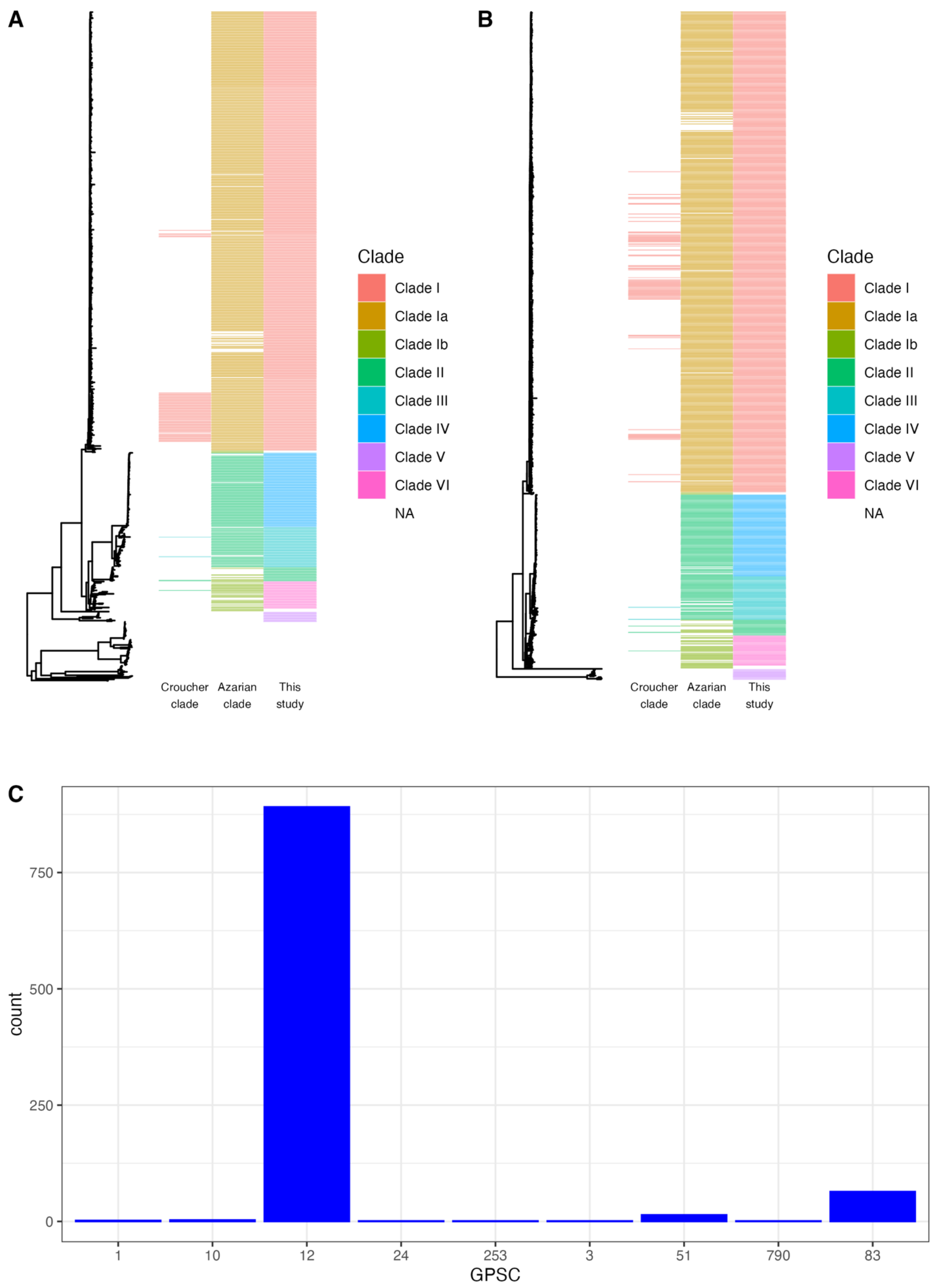
A
B
Clade
Clade I
Clade Ia
Clade Ib
Clade II
Clade III
Clade IV
Clade V
Clade VI
NA
Croucher clade
Azarian clade
This study
C
count
750
500
250
0
1
10
12
24
253
3
51
790
83
GPSC

Figure S2 Overview of the serotype 3 population. (A) Neighbour-joining tree calculated from core genome distances estimated using PopPUNK across the full serotype 3 dataset of 971 isolates. The columns show the assignment of GPSC12 isolates to clades in Croucher *et al* (2012); Azarian *et al* (2016), which was subsequently extended by Groves *et al* (2019); and this work. (B) Recombination-corrected maximum likelihood phylogeny of 891 GPSC12 isolates. The assignment to clades is shown as in panel A. (C) Bar chart showing the assignment of 971 serotype 3 isolates to GPSCs using PopPUNK and version 6 of the GPS strain database. The majority (891) isolates belonged to GPSC12.

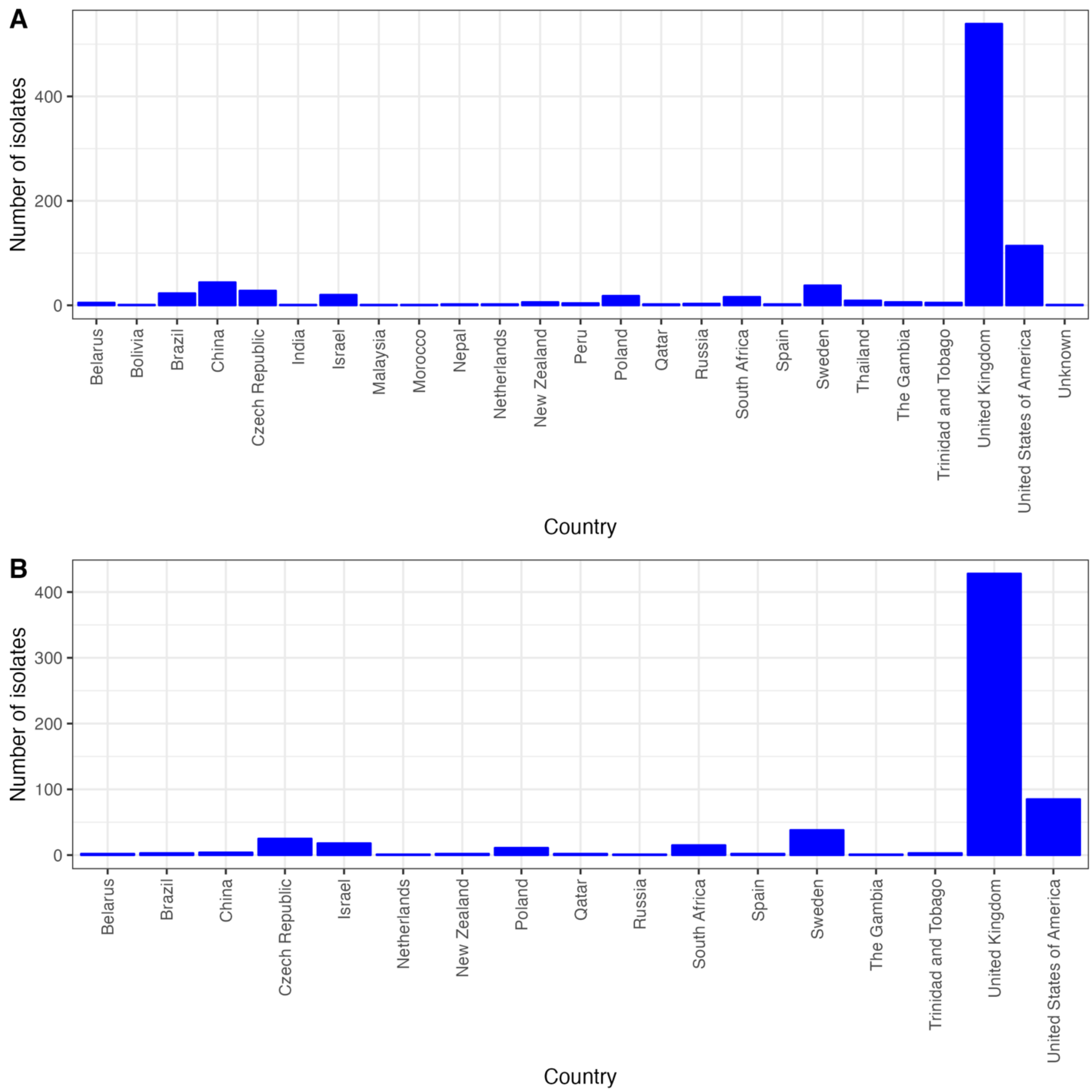

Figure S3 Geographic distribution of (A) the 891 GPSC12 isolates (B) the 641 Clade I isolates.

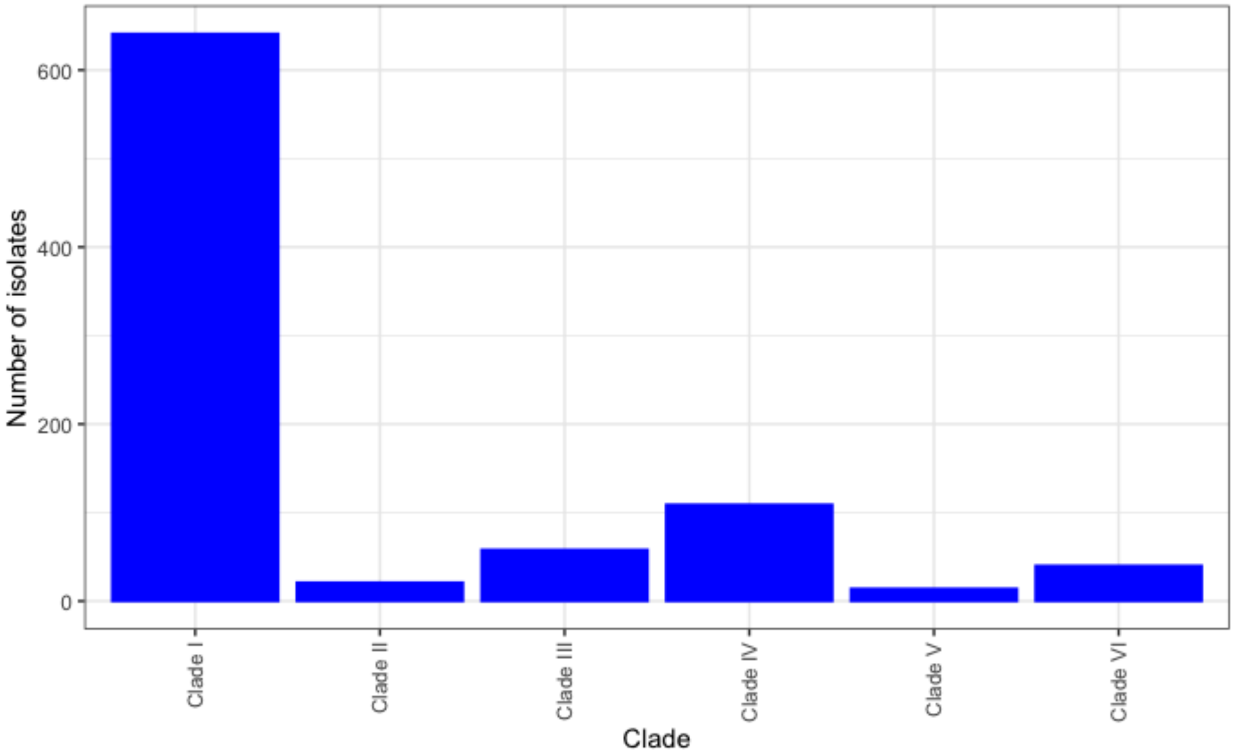

Figure S4 Distribution of the 891 GPSC12 isolates between the clades defined using the recombination-corrected phylogeny.

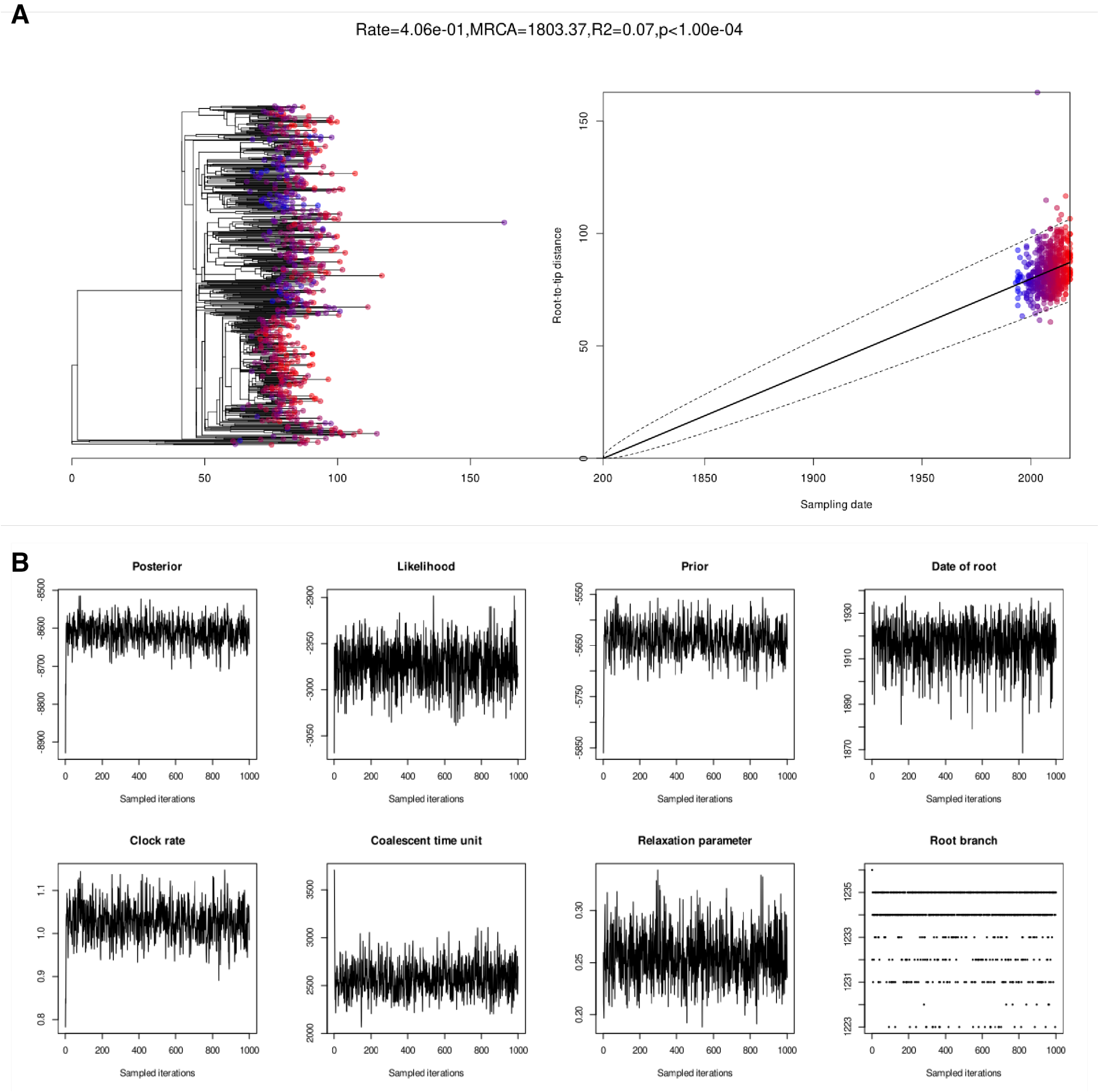

Figure S5 Phylodynamic analysis of Clade I with BactDating. (A) Root-to-tip distance analysis of Clade I. The left panel shows the Clade I subtree, extracted from the Gubbins analysis of GPSC12, rooted to maximise the correlation between the date at which isolates were sampled and their root-to-tip distance. Leaf nodes are coloured according to their sampling date: blue indicates bacteria collected relatively early, and red indicates bacteria collected relatively recently. The right panel shows the correlation between the sampling date and the root-to-tip distances, using the same colouring. The linear model fit shows the molecular clock rate, and the point at which the line intercepts the horizontal axis indicates the estimated date of Clade I's origin. (B) Markov Chain Monte Carlo sampling of the BactDating model parameters. These demonstrate the convergence of the algorithm on a stable set of parameter estimates.

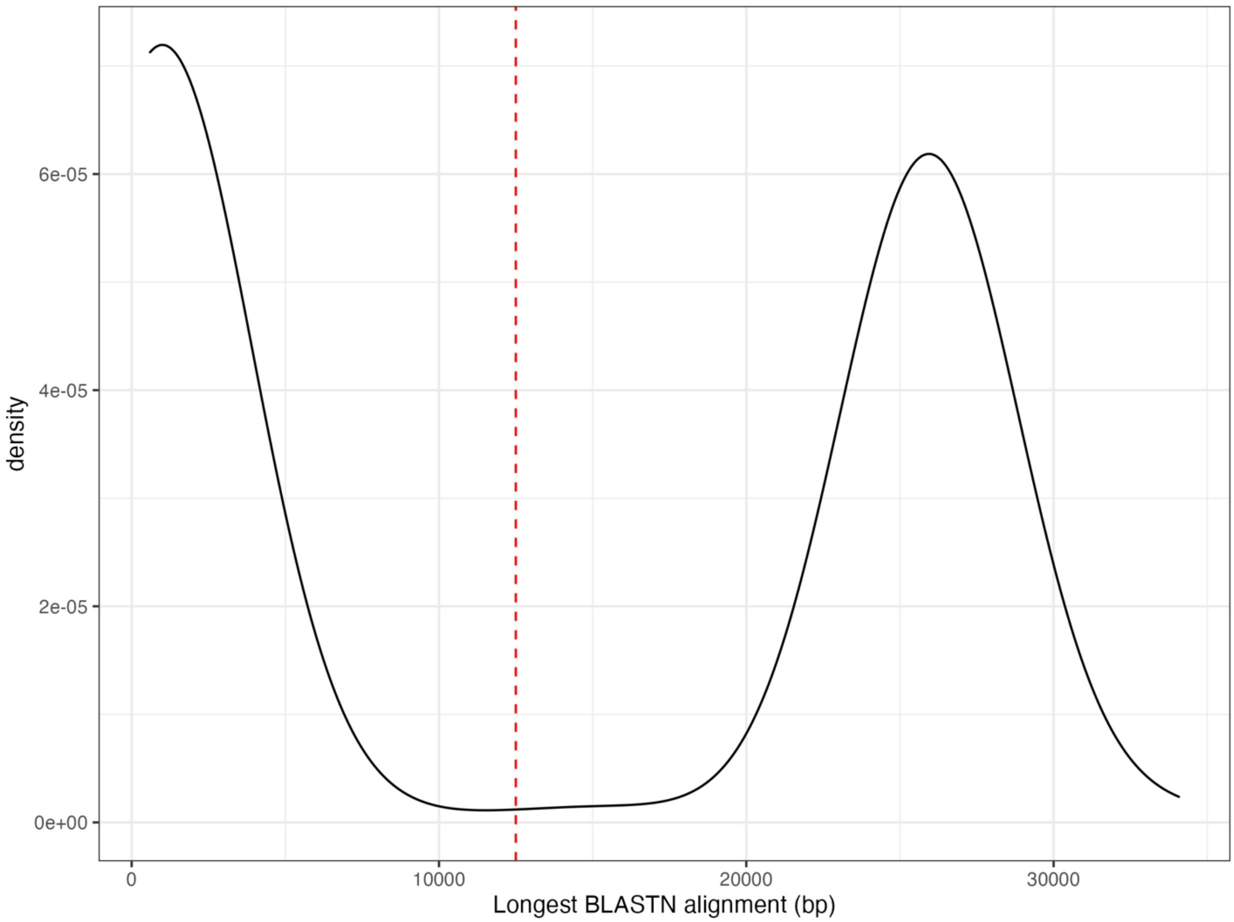

Figure S6 Inferring the distribution of ϕOXC141-like prophage. The 891 GPSC12 isolates were aligned to the 34,080 bp sequence of ϕOXC141, extracted from *S. pneumoniae* OXC141 (accession code FQ312027). The density plot shows the size distribution of the longest BLASTN alignment to the query sequence from each isolate. The red dashed line shows the threshold (12.5 kb) used to distinguish alignments to ϕOXC141-like prophage from less specific matches to more divergent prophage.

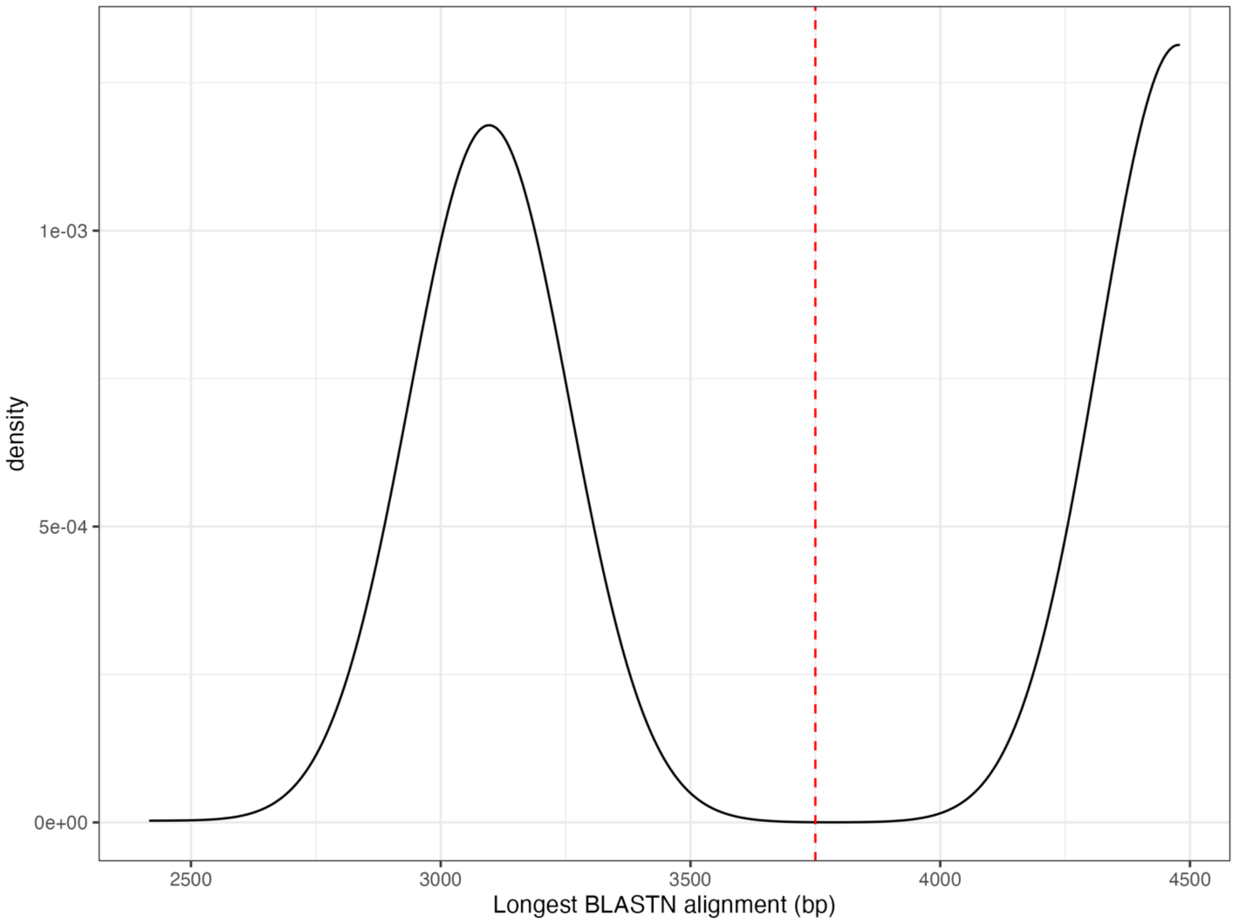

Figure S7 Inferring the status of the *attB*$_{OXC}$ site. The 891 GPSC12 isolates were aligned to a 4,477 bp sequence spanning the unmodified *attB*$_{OXC}$ insertion site within the *S. pneumoniae* TIGR4 genome (accession code AE005672). The density plot shows the size distribution of the longest BLASTN alignment to the query sequence from each isolate. The red dashed line shows the threshold (3,750 bp) used to distinguish the longer, unmodified *attB*$_{OXC}$ sites from those that are shorter, and therefore likely to have been disrupted by a prophage insertion.

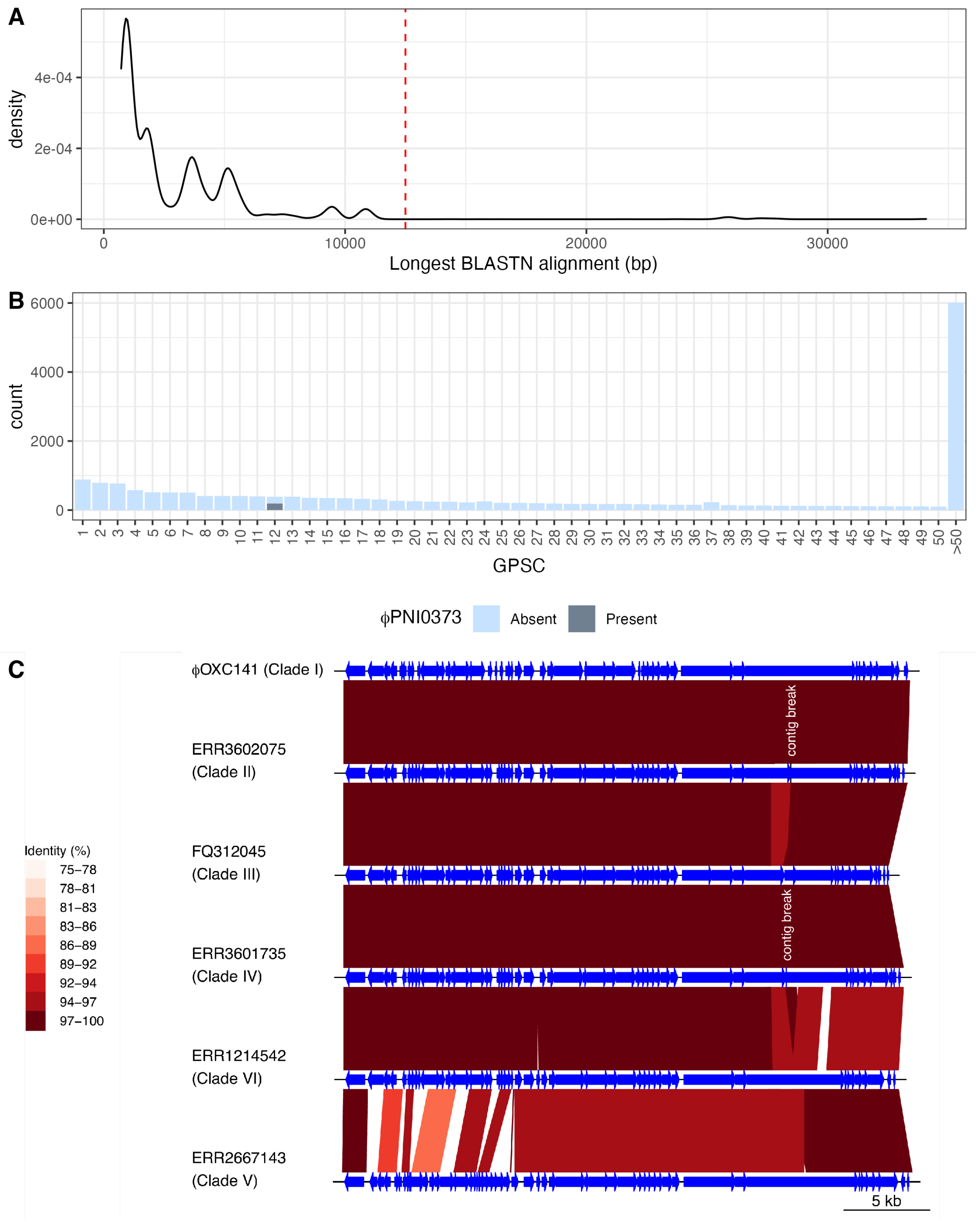

Figure S8 Identifying the distribution of prophage ϕOXC141 across the GPS collection. (A) The 34,080 bp sequence of ϕOXC141 was extracted from *S. pneumoniae* OXC141 (accession code FQ312027). The density plot shows the distribution of sizes for the longest BLASTN alignment to each GPS isolate. The red dashed line at 12.5 kb represents the threshold separating

alignments to full-length ϕOXC141-like prophage from matches to other phage sequences. (B) Distribution of ϕOXC141-like prophage between GPSCs using the 20,047 isolates in the GPS collection. GPSCs with an index above 50 are merged to enable visualisation of the prophage's enrichment in GPSC12. Evidence of ϕOXC141-like prophage was only identified in five isolates outside of GPSC12. (C) Comparison of ϕOXC141-like prophage in GPSC12 isolates outside of Clade I. The blue arrows represent predicted protein coding sequences. The red bands between prophage link regions of similar sequence, with the level of DNA sequence identity calculated using BLASTN indicated by the key. Each prophage is labelled with the accession code of the isolate in which it was identified, and the clade to which the isolate was assigned. Two prophage did not assemble within a single contig, and therefore the contig breaks are marked on the sequences.

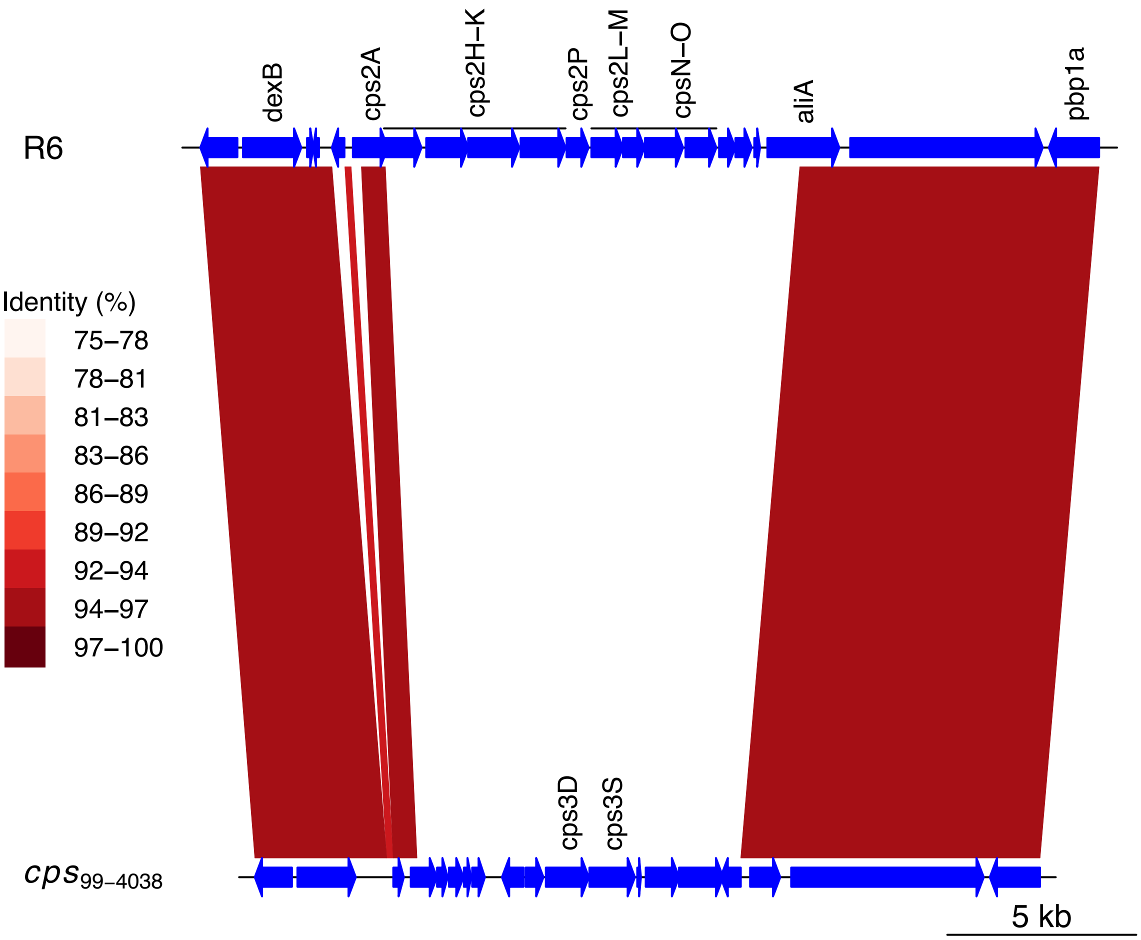

Figure S9 Replacement of the *cps* locus of *S. pneumoniae* R6 with that from *S. pneumoniae* 99-4038 in the recombinant *S. pneumoniae cps*$_{99\text{-}4038}$. The region encompassed by the recombination inferred to span the *cps* locus in *S. pneumoniae cps*$_{99\text{-}4038}$ is aligned to the orthologous region of the parental genotype, R6, with BLASTN. The red bands link regions of similar sequence, with the colour indicating the level of sequence identity between the pair. The *cps* locus itself is flanked by the *dexB* and *aliA* genes. The serotype 3 allele contains two functional genes: *cps3D*, encoding a UDP-glucose 6-dehydrogenase, and *cps3S*, encoding the polymerase.

A

B

C

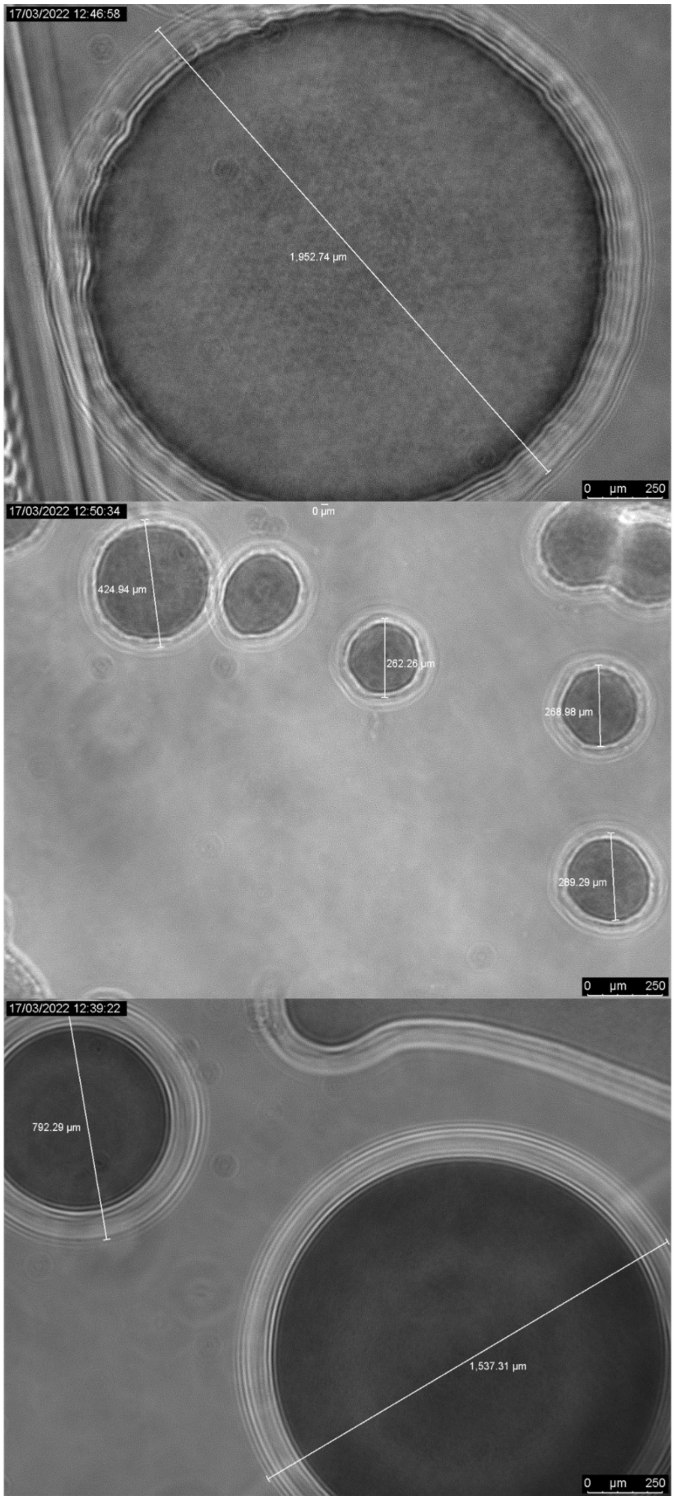

Figure S10 Light microscopy of pneumococcal colonies. (A) Large mucoid colonies of *S. pneumoniae* 99-4038, a serotype 3 isolate. (B) Small colonies of the unencapsulated *S. pneumoniae* R6. (C) Large mucoid colonies of *S. pneumoniae* R6 $cps_{99\text{-}4038}$, engineered to express the serotype 3 capsule.

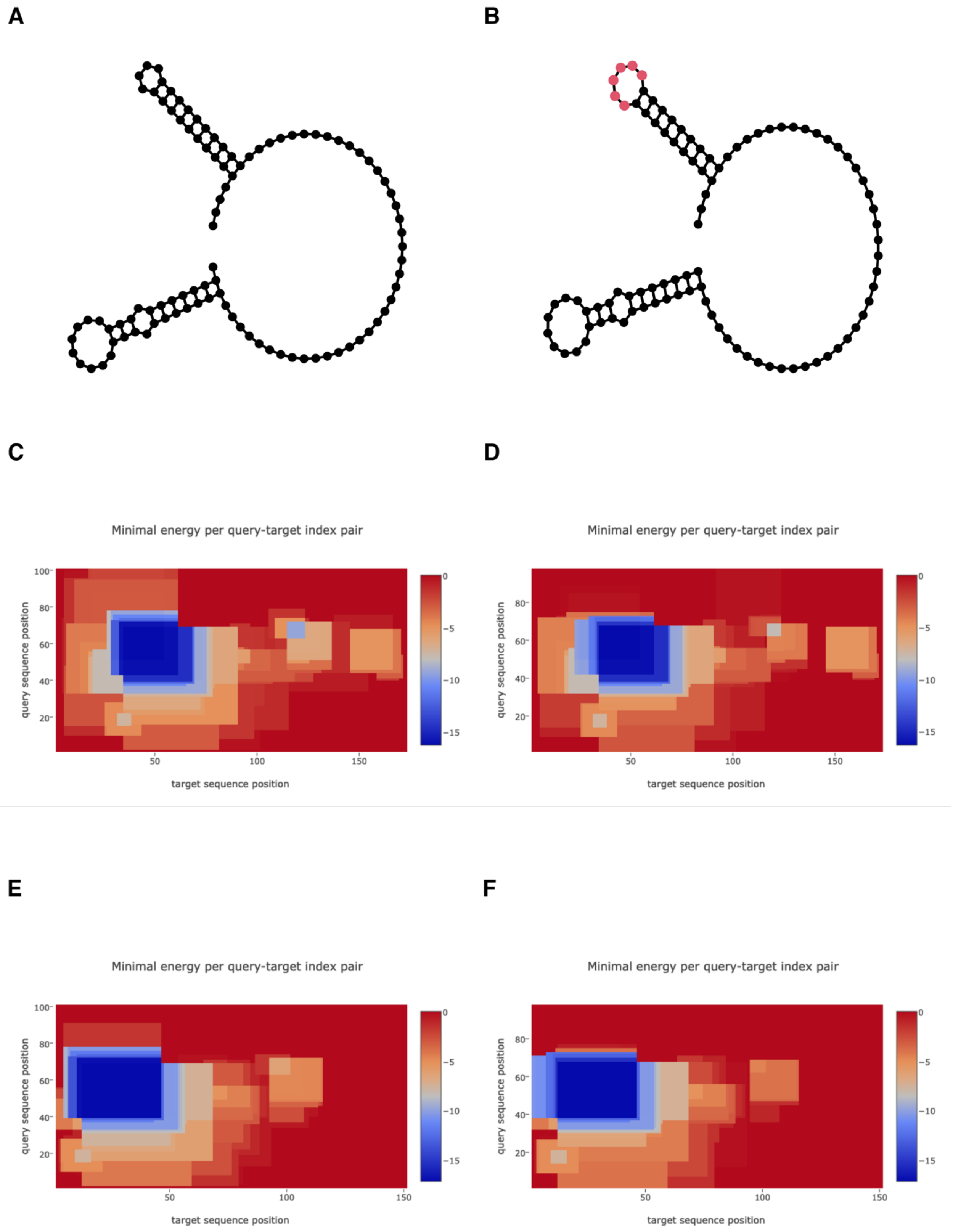
A
B
C
D
Minimal energy per query-target index pair
query sequence position
target sequence position
0
−5
−10
−15
20
40
60
80
100
50
100
150
E
F
Minimal energy per query-target index pair
query sequence position
target sequence position

Figure S11 Comparison of the predicted structures of (A) csRNA3 from *S. pneumoniae* R6 and (B) csRNA3L from *S. pneumoniae* OXC141. The sequence "CAAUCA" is highlighted in red. (C) Predicted interaction between csRNA3 from *S. pneumoniae* R6 and the *comC* transcript from *S. pneumoniae* OXC141, which has the CSP1 pherotype. The horizontal axis corresponds to the length of the *comC* mRNA (from the transcription initiation site to the stop codon of the protein coding sequence), and the vertical axis corresponds to the length of csRNA3. The colour shows the minimal energy of the interaction between the sequences at the coordinates specified by the axes in kcal mol^{-1}, as indicated by the key. (D) Predicted interaction between csRNA3L from *S. pneumoniae* OXC141 and the *comC* transcript from *S. pneumoniae* OXC141, which has the CSP1 pherotype. Data are displayed as described for panel (C). (E) Predicted interaction between csRNA3 from *S. pneumoniae* R6 and the *comC* transcript from *S. pneumoniae* RMV8, which has the CSP2 pherotype. Data are displayed as described for panel (C). (F) Predicted interaction between csRNA3L from *S. pneumoniae* OXC141 and the *comC* transcript from *S. pneumoniae* RMV8, which has the CSP2 pherotype. Data are displayed as described for panel (C).

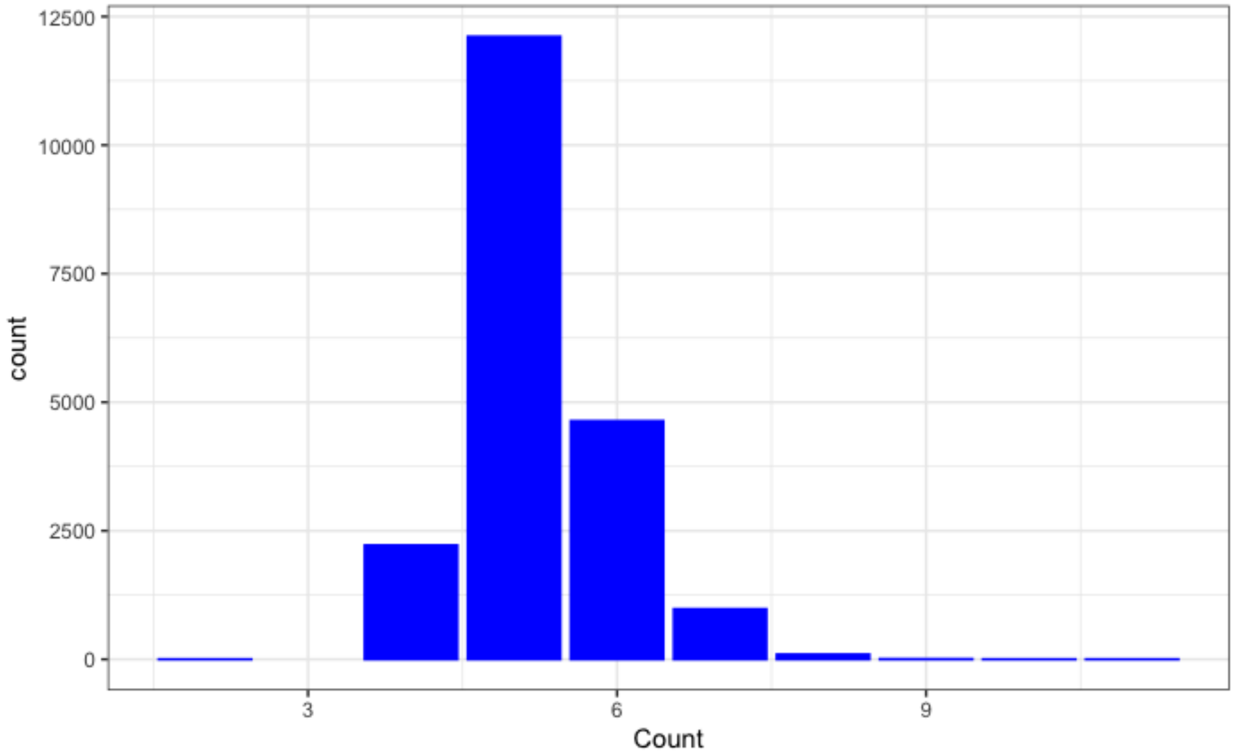

Figure S12 Distribution of csRNA sequences across GPS isolates. The bar chart shows the number of csRNA sequences identified in each of the 20,047 GPS isolates.

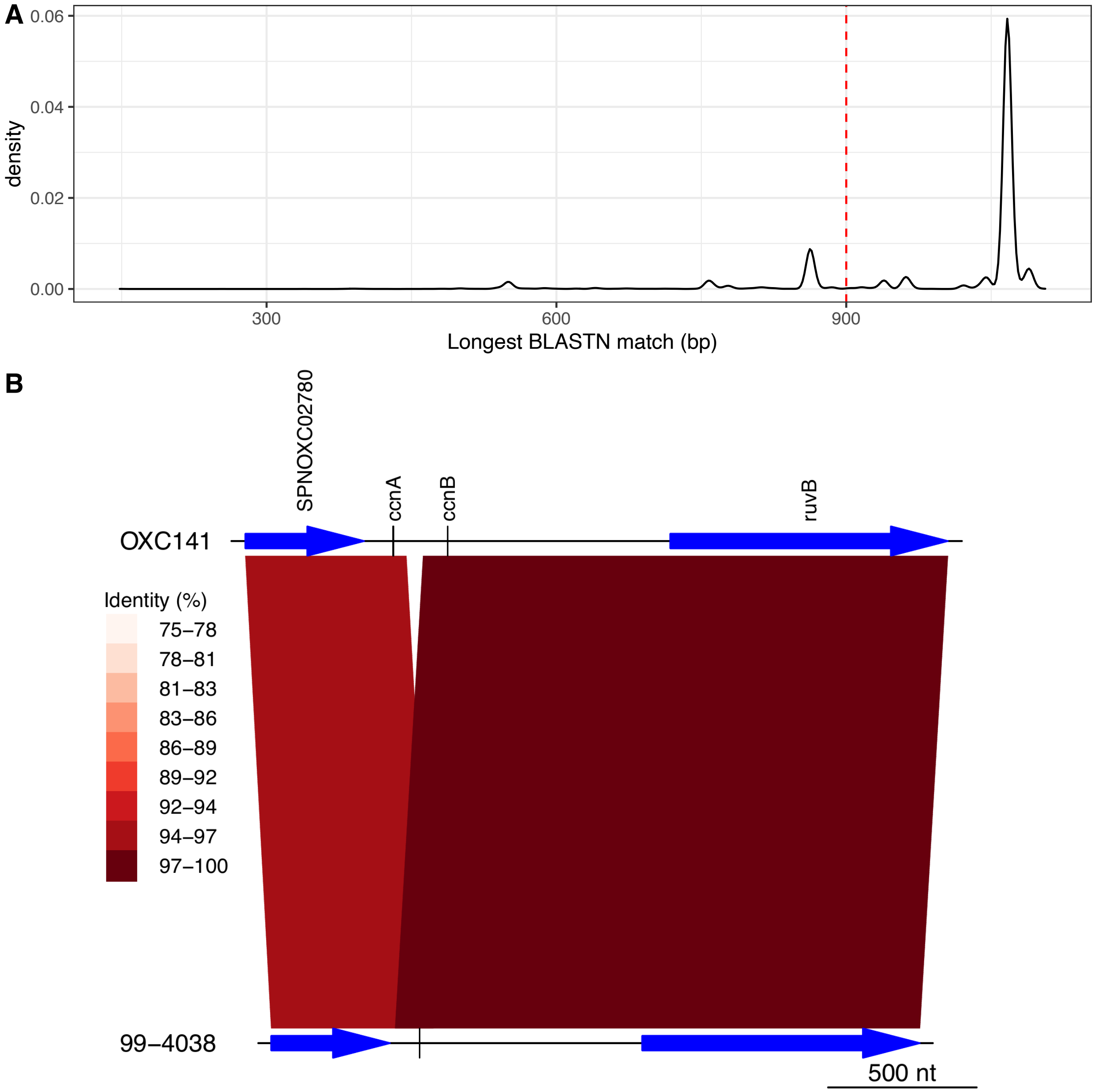

Figure S13 Identifying deletions affecting *ccnA* and *ccnB*. (A) A 1,067 bp sequence spanning the region upstream of *ruvB*, which encodes *ccnA* and *ccnB*, was extracted from *S. pneumoniae* R6 and aligned to the 20,047 GPS isolates. The density plot shows the distribution of sizes for the longest BLASTN alignment to each GPS isolate. The red dashed line at 900 bp represents the threshold separating the full-length loci from those having undergone a deletion in this part of the genome. (B) Formation of a chimeric *ccnAB* gene through an intragenomic recombination between *ccnA* and *ccnB*. The complete genomes of Clade I isolates *S. pneumoniae* OXC141 and 99-4038 were aligned with BLASTN. The blue arrows show protein coding sequences, and the black vertical lines mark the non-coding csRNA genes. The red bands link regions of similar sequence. The colours of the bands indicate the level of similarity between the sequences. The V-shape of the bands shows how the tandem csRNA genes at this locus have recombined and deleted the intervening sequence, generating *ccnAB* within *S. pneumoniae* 99-4038.

A

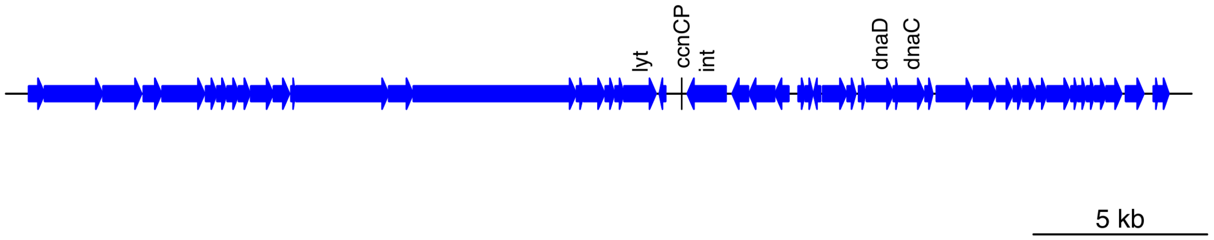

B

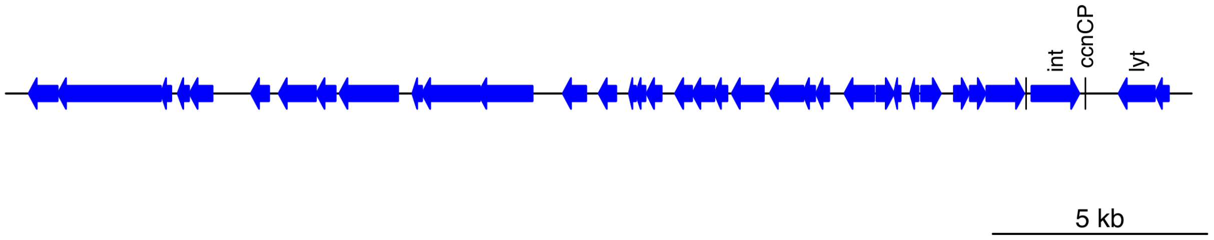

Figure S14 Identification of *ccnCP* on pneumococcal prophage sequences. (A) Annotation of the prophage *S. pneumoniae* SpSL1 (accession code KM882824), showing the presence of *ccnCP* between the *int* and *lyt* genes, corresponding to the *attP* site of the phage. (B) Annotation of the prophage *S. pneumoniae* ϕARI0578 (accession code KT337360), showing the presence of *ccnCP* between the *int* and *lyt* genes, corresponding to the *attP* site of the phage.

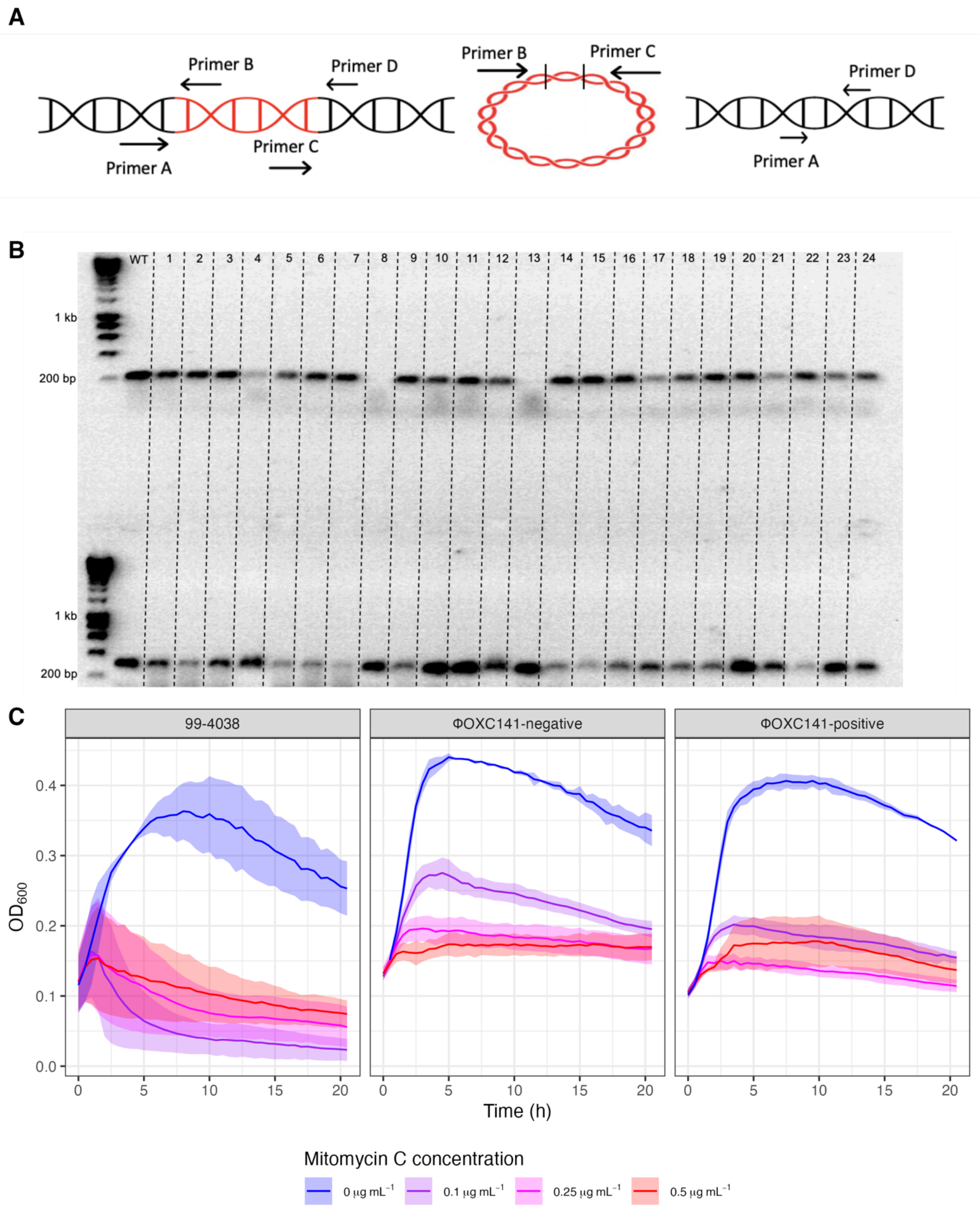

Figure S15 Isolation of *S. pneumoniae* 99-4038 ΔϕOXC141. (A) Schematic showing how four primers were used to determine the distribution and activity of prophage. Primers A and B were used to detect the $attL_{\mathrm{OXC}}$ site of an integrated prophage; primers B and C were used to detect the $attP_{\mathrm{OXC}}$ site on circular excised prophage; and primers A and D were used to detect the

attB$_{OXC}$ site in bacteria in which the prophage has excised or been deleted. (B) Agarose gel showing the A and B, and A and D, PCR products for the wild-type 99-4038 genotype (WT) and 24 colonies isolated following three rounds of mitomycin C exposure in a passage experiment. The size markers are a HyperLadder 1kb (Bioline). The top row shows the products of primers A and B (expected size of 217 bp), indicating the presence of an integrated ϕOXC141. The bottom row shows the products of primers A and D (expected product size of 307 bp), indicating the presence of an intact *attB*$_{OXC}$ site resulting from prophage excision or deletion. Each column corresponds to a different isolate; those numbered 8 and 13 were confirmed to have the ΔϕOXC141 genotype by further PCR experiments. (C) Mitomycin C sensitivity of the parental *S. pneumoniae* 99-4038 isolate, and ϕOXC141-negative and ϕOXC141-positive isolates from the third round of the passage. The isolates found to be ϕOXC141-negative by PCR amplification also exhibited higher tolerance of mitomycin C than those that were found to be ϕOXC141-positive. This would be expected if the prophage has been deleted, rather than excised.

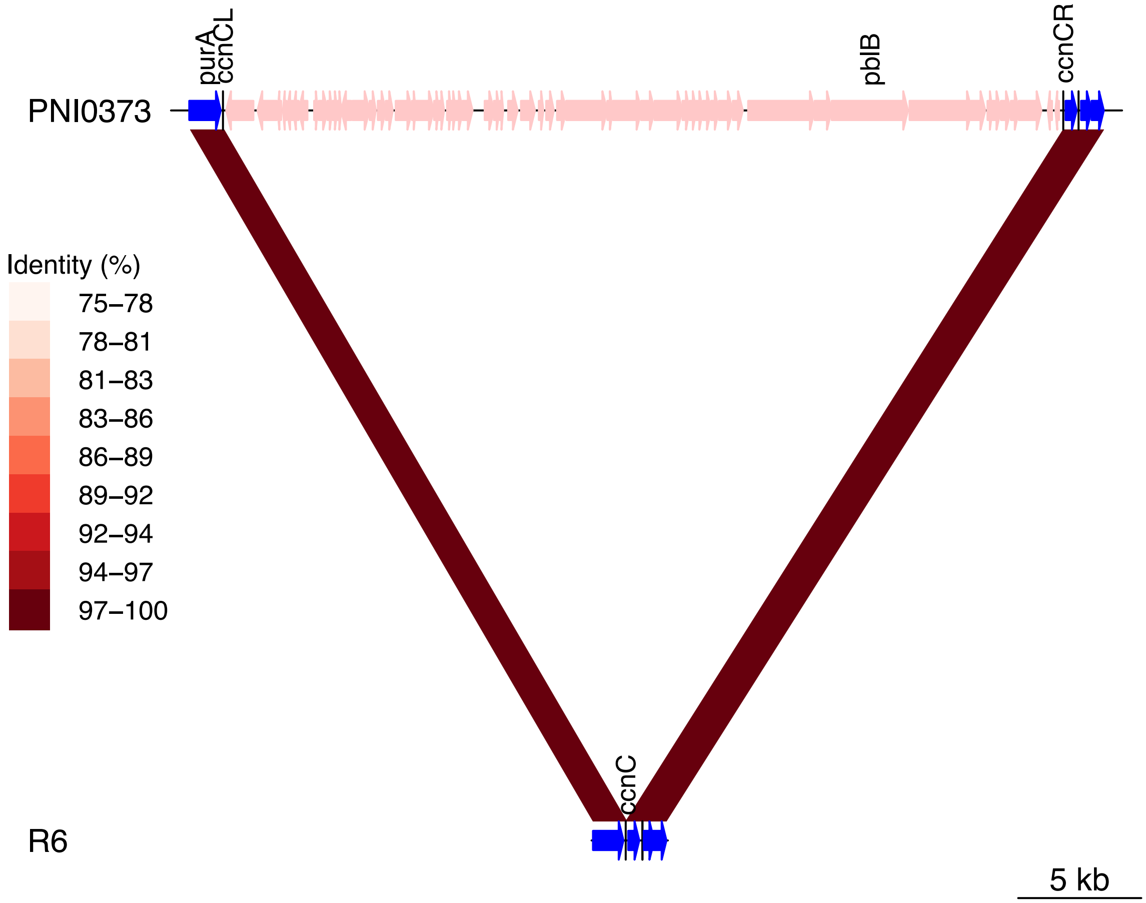

Figure S16 Modification of *ccnC* through insertion of ϕPNI0373 into *attB*$_{\mathrm{OXC}}$. The insertion of ϕPNI0373, indicated by the pink genes integrated downstream of *purA*, into the *S. pneumoniae* PNI0373 genome (accession code CP001845) is compared to the unmodified *attB*$_{\mathrm{OXC}}$ site of R6 using BLASTN. The red bands link regions of similar sequence, with the colour indicating the level of sequence identity between the pair. The black vertical lines show how the cellular *ccnC* gene at the *attB* site is split into the *ccnCL* and *ccnCR* genes at the *attL* and *attR* sites, respectively.

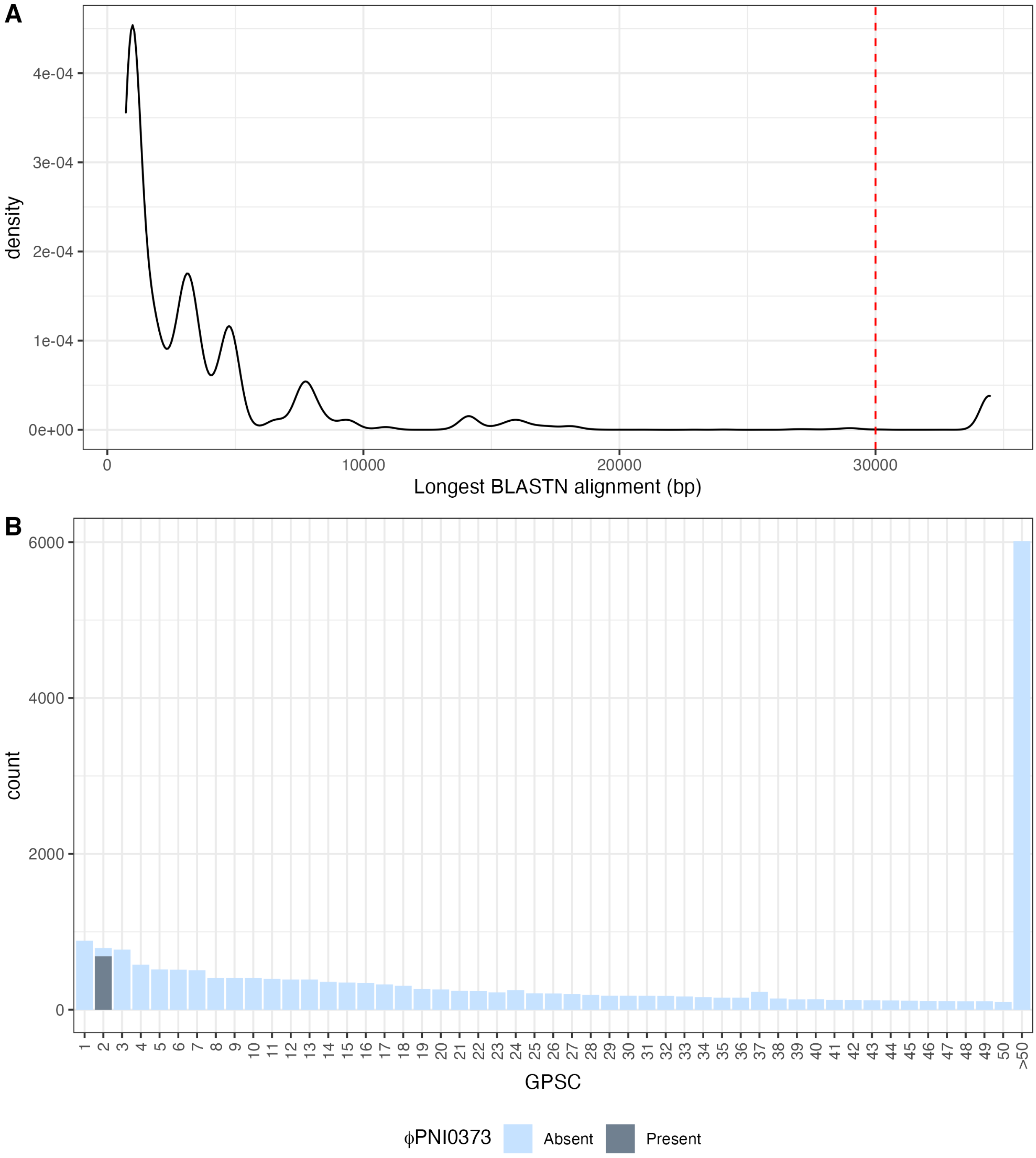

Figure S17 Identifying the distribution of prophage ϕPNI0373. (A) The 34,437 bp sequence of ϕPNI0373 was extracted from *S. pneumoniae* PNI0373 (accession code CP001845). The density plot shows the distribution of sizes for the longest BLASTN alignment to each GPS isolate. The red dashed line at 30 kb represents the threshold separating alignments to full-length ϕPNI0373-like prophage from matches to other phage sequences. (B) Distribution of ϕPNI0373-like prophage between GPSCs using the 20,047 isolates in the GPS collection. GPSCs with an index above 50 are merged to enable visualisation of the prophage's enrichment in GPSC2. No evidence was found of ϕPNI0373-like prophage outside of GPSC2.

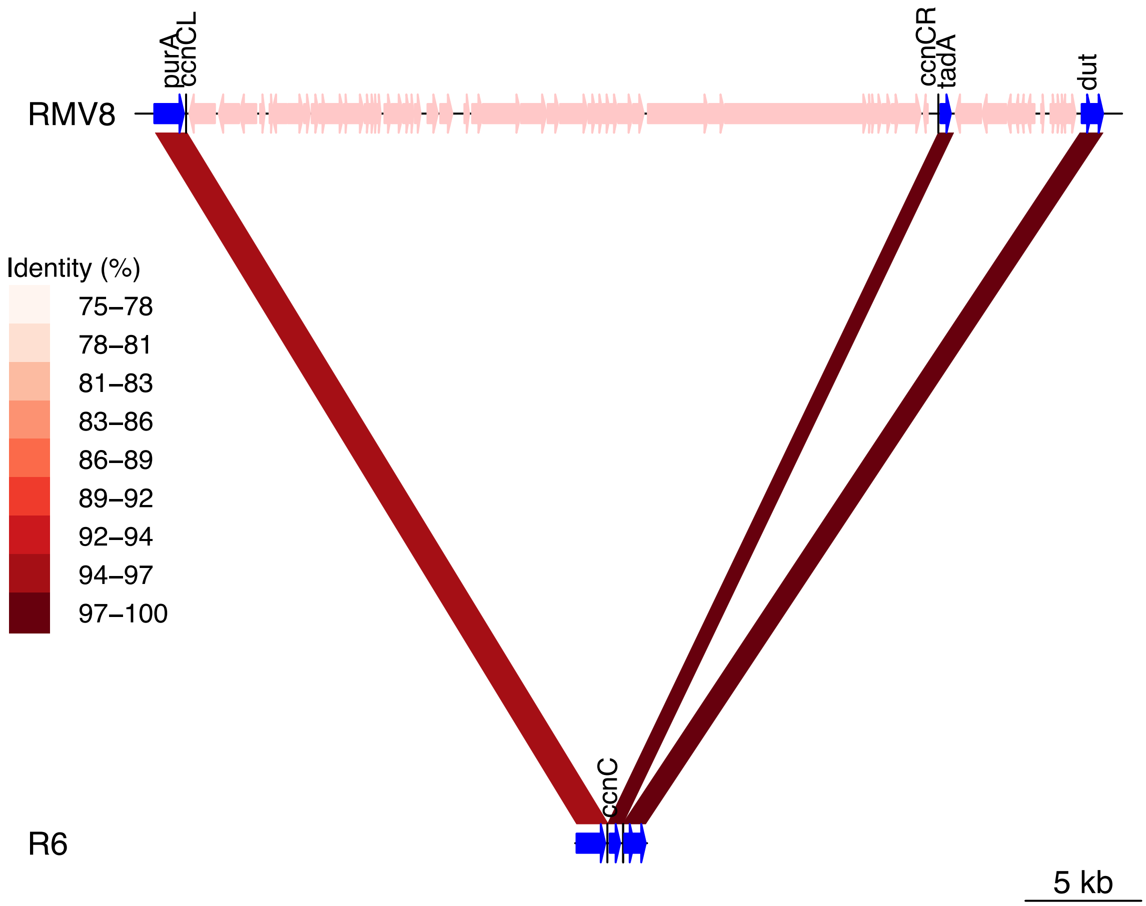

Figure S18 Modification of *ccnC* through insertion of ϕRMV8 into *attB*$_{OXC}$. The insertion of ϕRMV8, indicated by the pink genes integrated downstream of *purA*, into the *S. pneumoniae* RMV8 genome (accession code OX244288) is compared to the unmodified *attB*$_{OXC}$ site of R6 using BLASTN. The red bands link regions of similar sequence in the two loci, with the colour indicating the level of sequence identity between the pair. The black vertical lines show how the cellular *ccnC* gene at the *attB* site is split into the *ccnCL* and *ccnCR* genes at the *attL* and *attR* sites, respectively. The second set of pink genes, inserted on the right of ϕRMV8, represents a phage-related chromosomal island (PRCI) inserted adjacent to the *tadA* gene.

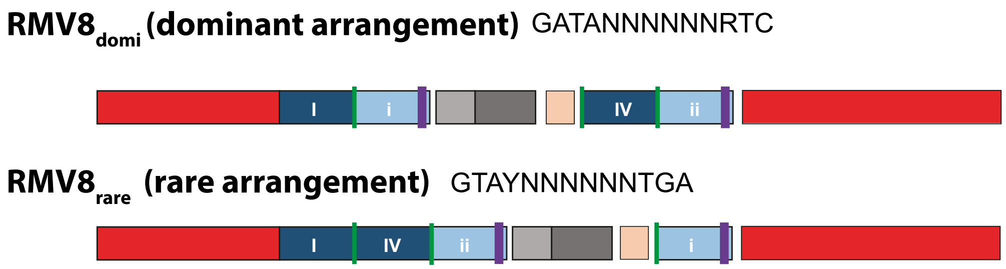

Figure S19 Differential arrangement of the *tvr* loci distinguishing $RMV8_{domi}$ and $RMV8_{rare}$. Each is labelled with the DNA motif they target. The red boxes correspond to the conserved methylase (*hsdM*, at the 5' end) and endonuclease (*hsdR*, at the 3' end) genes. The blue boxes correspond to the sequences encoding the target-recognition domains (TRDs) of the specificity subunit. The dark blue boxes correspond to the 5' TRD-encoding sequences, and the light blue boxes correspond to the 3' TRD-encoding sequences. The active specificity subunit gene (*hsdS*) is formed by the combination of a 5' and 3' TRD-encoding sequence that is closest to the 5' end of the locus. The HsdS protein determines the motif targeted by the system encoded by the *tvr* locus. The rearrangement of the TRD-encoding sequences is driven by TvrR, a recombinase encoded by a gene represented by the pink box, which is regulated by the products of the *tvrAT* genes, represented by the grey boxes. The *tvrR* gene is truncated in these “locked” variants to limit the interconversion between arrangements at the *tvr* locus.

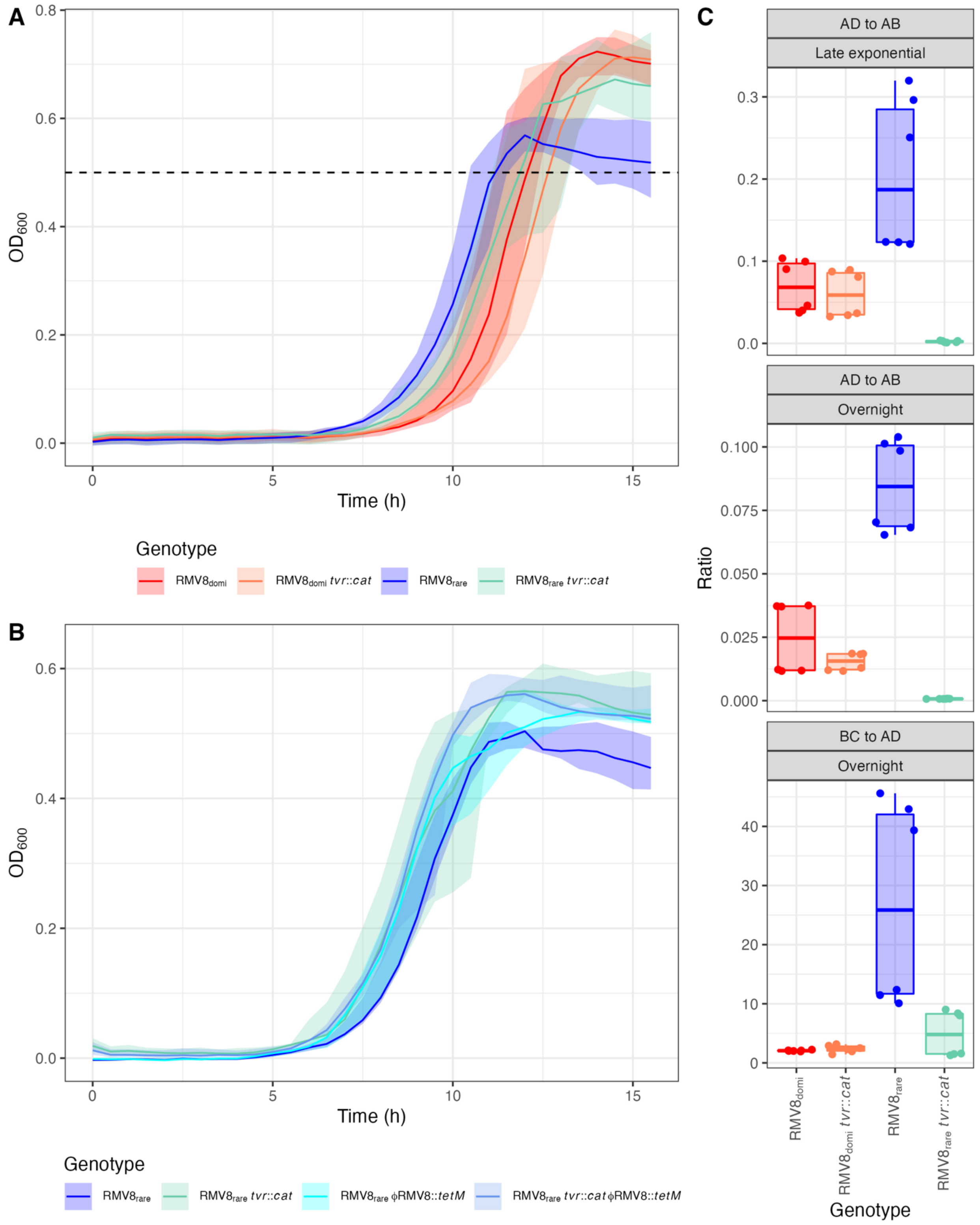

Figure S20 Growth characteristics of RMV8 genotypes. (A) Growth curves of *S. pneumoniae* RMV8 genotypes analysed by RNA-seq. The solid line shows the median, and the shaded region shows the minimum to maximum range, from eight replicate experiments for each genotype. The atypical growth profile is that of *S. pneumoniae* RMV8$_{rare}$, which has a growth

defect in late exponential phase, relative to other genotypes. The dashed horizontal line at an OD_{600} of 0.5 corresponds to the cell density at which samples for RNA-seq were taken for each genotype. (B) Growth curves for $RMV8_{rare}$ genotypes. Data are shown as in panel (A), but summarise four replicate experiments per genotype. The growth defect of $RMV8_{rare}$ is eliminated by the replacement of prophage ϕRMV8 with a *tetM* resistance marker. (C) Assays of the activity of ϕRMV8 using quantitative PCR (qPCR). The same primer arrangements are used as displayed in Additional file 1: Fig. S15. The proportion of phage excised from the chromosome was estimated by qPCR of an amplicon spanning the *attL* site (using primers A and B), the *attB* site (using primers A and D), and the *attP* site (using primers B and C). The ratios of the concentrations of the products of primers A and D and primers A and B were used to quantify the level of prophage excision for the four genotypes analysed by RNA-seq during the late exponential phase (OD_{600} = 0.5), and after overnight growth. The ratios of the concentrations of the products of primers B and C and the products of primers A and D were used to quantify the level of circularised phage for the same four RMV8 genotypes after overnight growth.

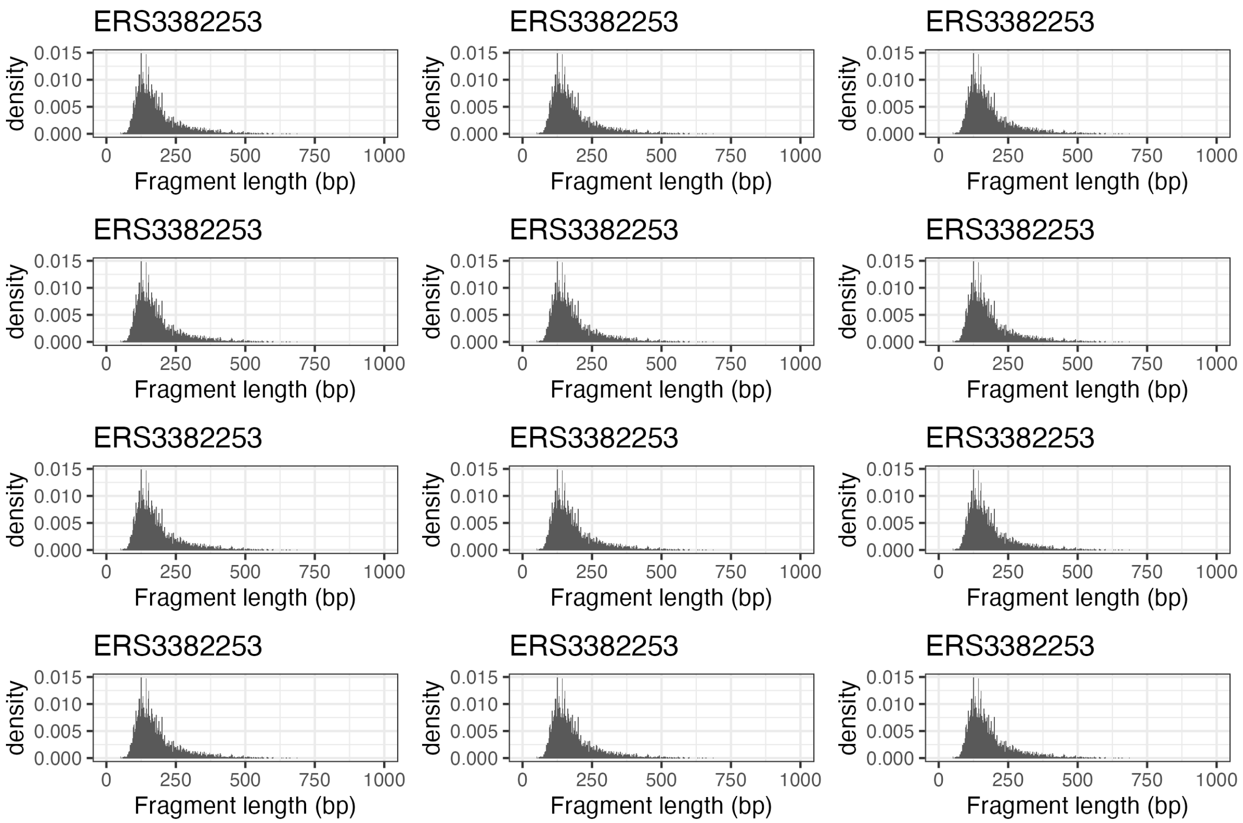

Figure S21 Density plots showing the inferred distribution of fragment sizes from mapping by Kallisto. The consistency of these distributions between samples, each labelled with its accession code (Additional file 5: Table S4), suggests there should not be any bias introduced by variation in sequencing library preparation.

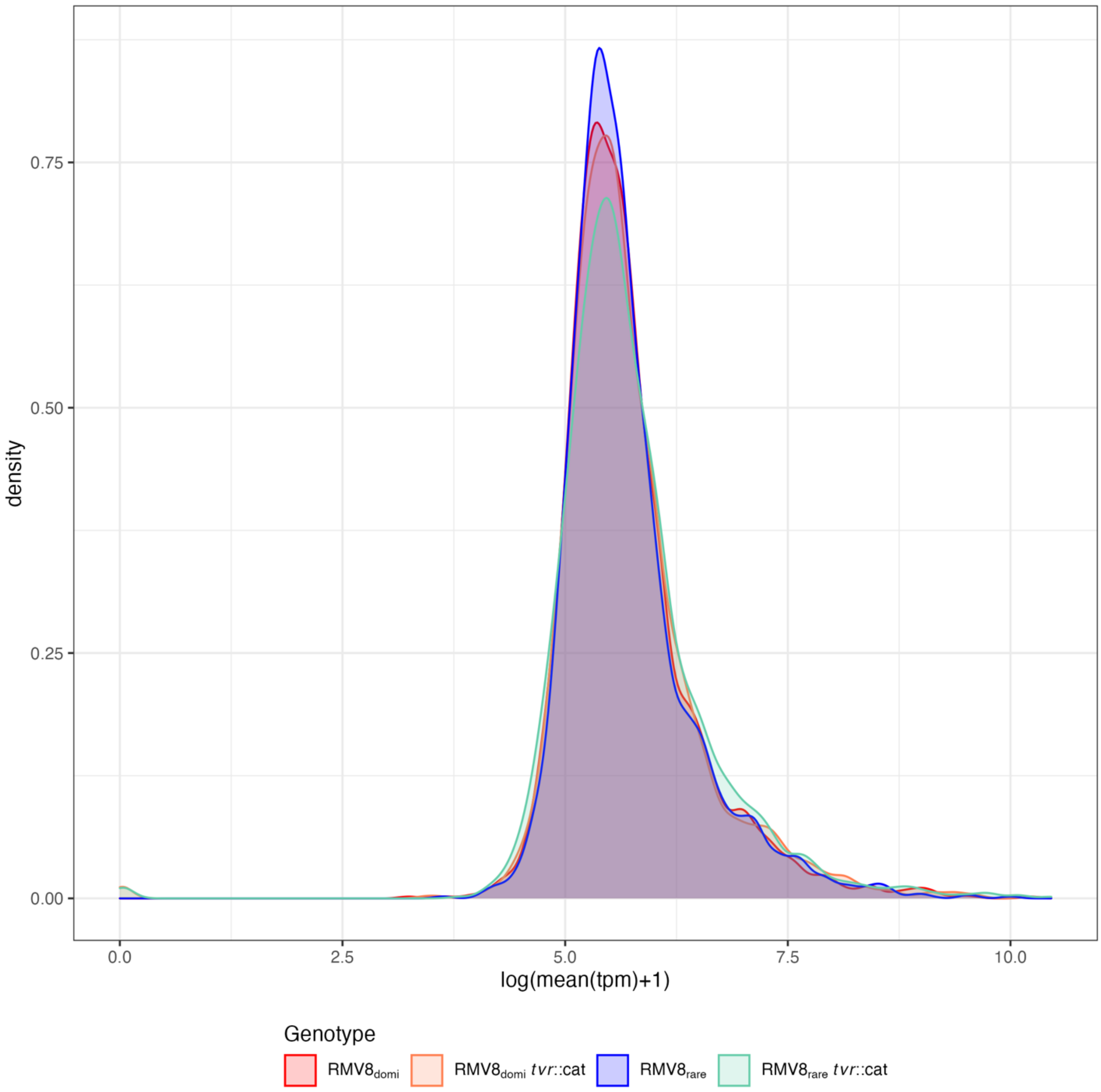

Figure S22 Density plots showing the distribution of mean transcripts per million (tpm) values across coding sequences for samples in different groups. The consistency of these distributions suggests there should not be any systematic biases causing false positive inferences of transcriptional differences between samples.

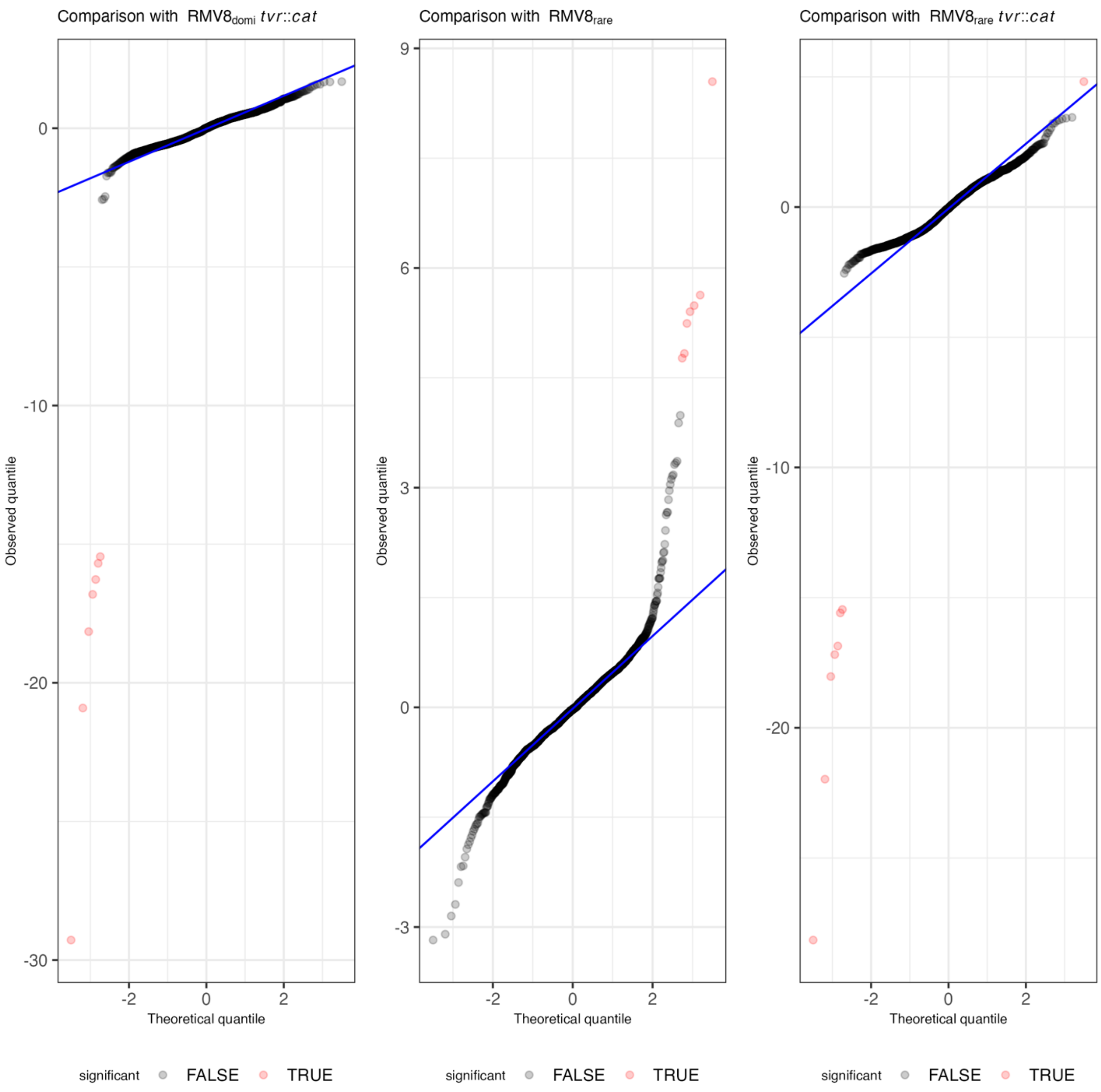

Figure S23 Q-Q plots comparing the theoretical and observed distributions of the Wald test statistic across genes for the contrast of transcriptional patterns between $RMV8_{domi}$ and the other analysed genotypes, as indicated by the plot titles. The blue line shows the relationship expected under the null hypothesis of no difference in expression patterns. Each point represents a coding sequence. Points are coloured red if the null hypothesis can be rejected at a false discovery rate of 10^{-3}, following a Benjamini-Hochberg correction for multiple testing. The Q-Q plot shows this threshold captures the major differences between the genotypes.

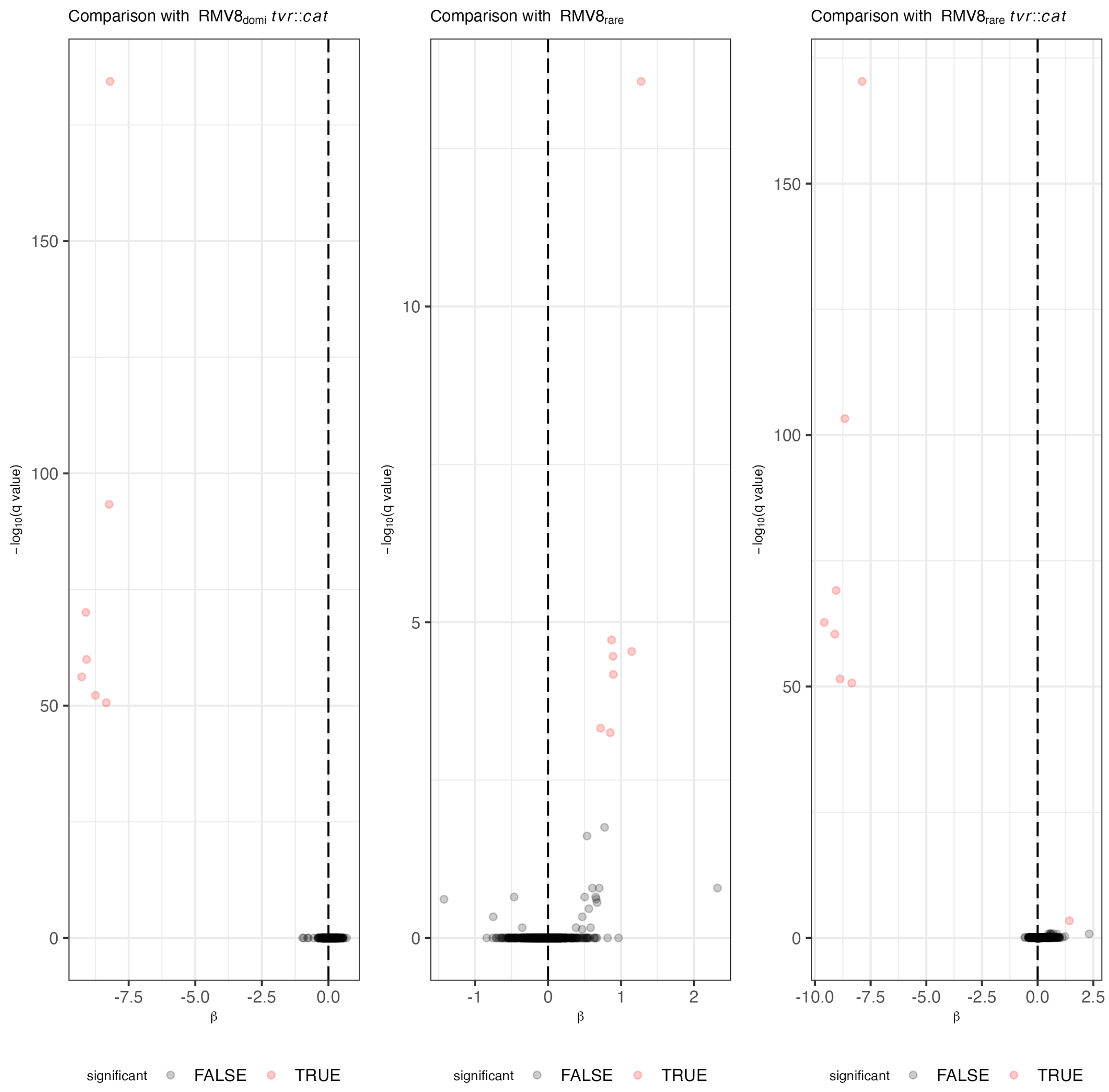

Figure S24 Volcano plots contrasting the transcriptional patterns between $RMV8_{domi}$ and the other analysed genotypes, as indicated by the plot titles. The horizontal axis shows the natural logarithm of the fold difference in expression levels between the genotypes, β. The vertical axis shows the negative base 10 logarithm of the *q* value, calculated using a Benjamini-Hochberg correction. Points are coloured red where this value exceeds the false discovery rate threshold of 10^{-3}.

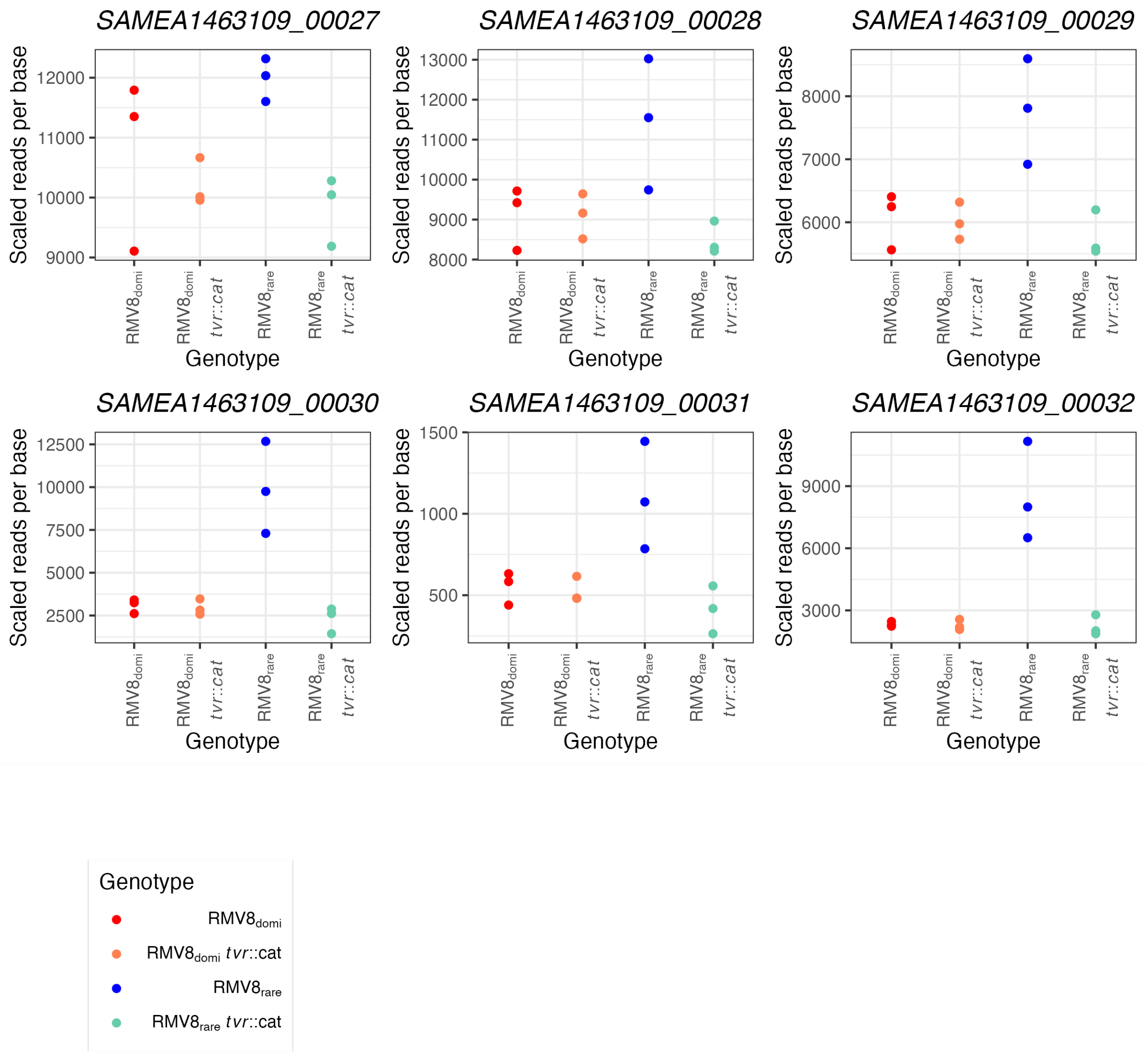

Figure S25 Quantification of the expression of ϕRMV8 lysogeny genes using RNA-seq data. Points are coloured using the scheme described in Fig. 6, as shown in the key. Each plot shows the transcription of a different gene across the four genotypes in scaled reads per base, which represents gene expression as the normalised mean number of reads mapping to the bases in a sequence. These genes tend to be most highly expressed in $RMV8_{rare}$.

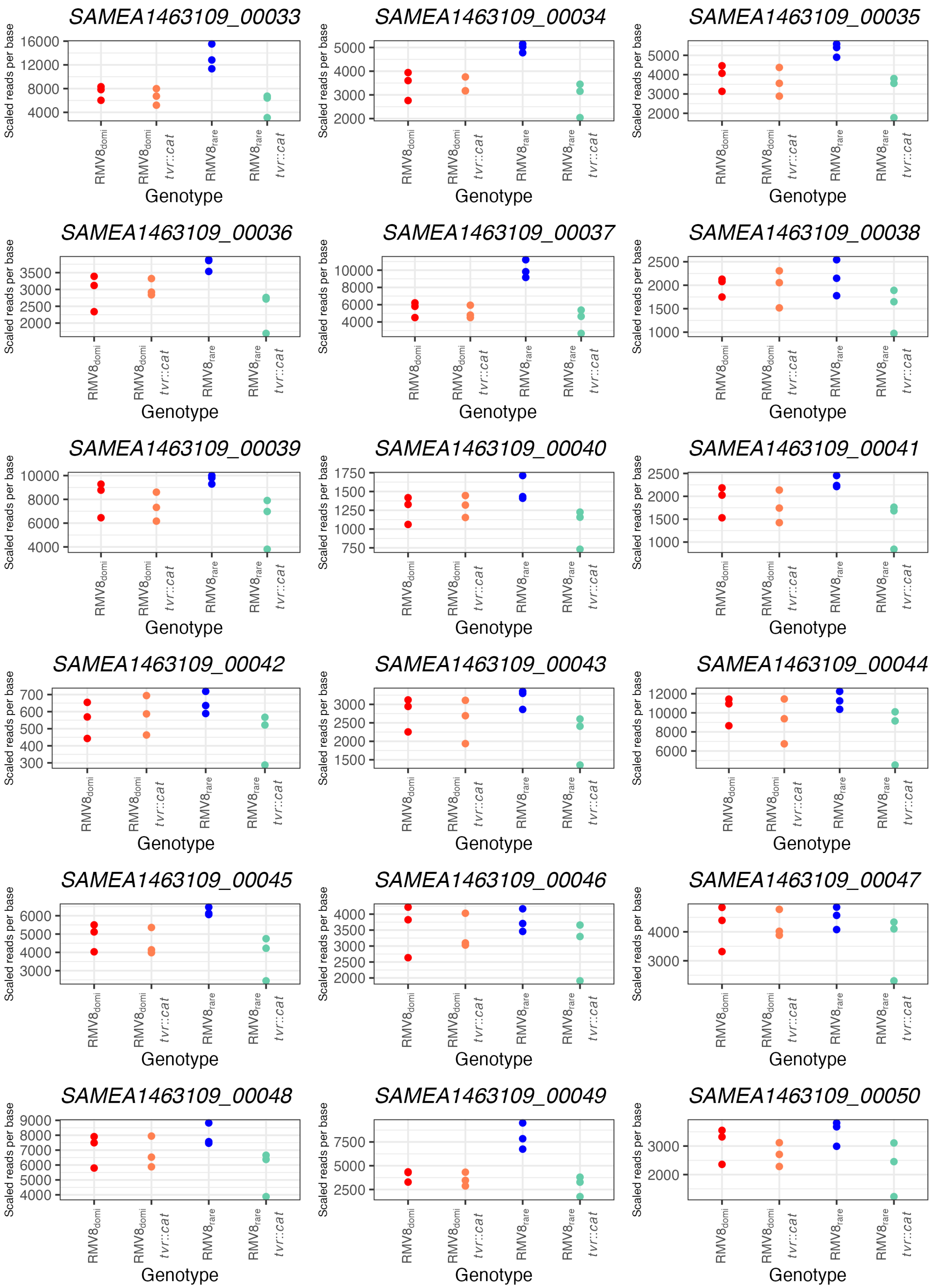
SAMEA1463109_00033
SAMEA1463109_00034
SAMEA1463109_00035
SAMEA1463109_00036
SAMEA1463109_00037
SAMEA1463109_00038
SAMEA1463109_00039
SAMEA1463109_00040
SAMEA1463109_00041
SAMEA1463109_00042
SAMEA1463109_00043
SAMEA1463109_00044
SAMEA1463109_00045
SAMEA1463109_00046
SAMEA1463109_00047
SAMEA1463109_00048
SAMEA1463109_00049
SAMEA1463109_00050
Scaled reads per base
RMV8domi
RMV8domi tvr::cat
RMV8rare
RMV8rare tvr::cat
Genotype

Figure S26 Quantification of the expression of ϕRMV8 replication genes using RNA-seq data. Points are coloured using the scheme described in Fig. 6. Each plot shows the transcription of a different gene, in scaled reads per base, across the four genotypes. These genes tend to be most highly expressed in $RMV8_{rare}$.

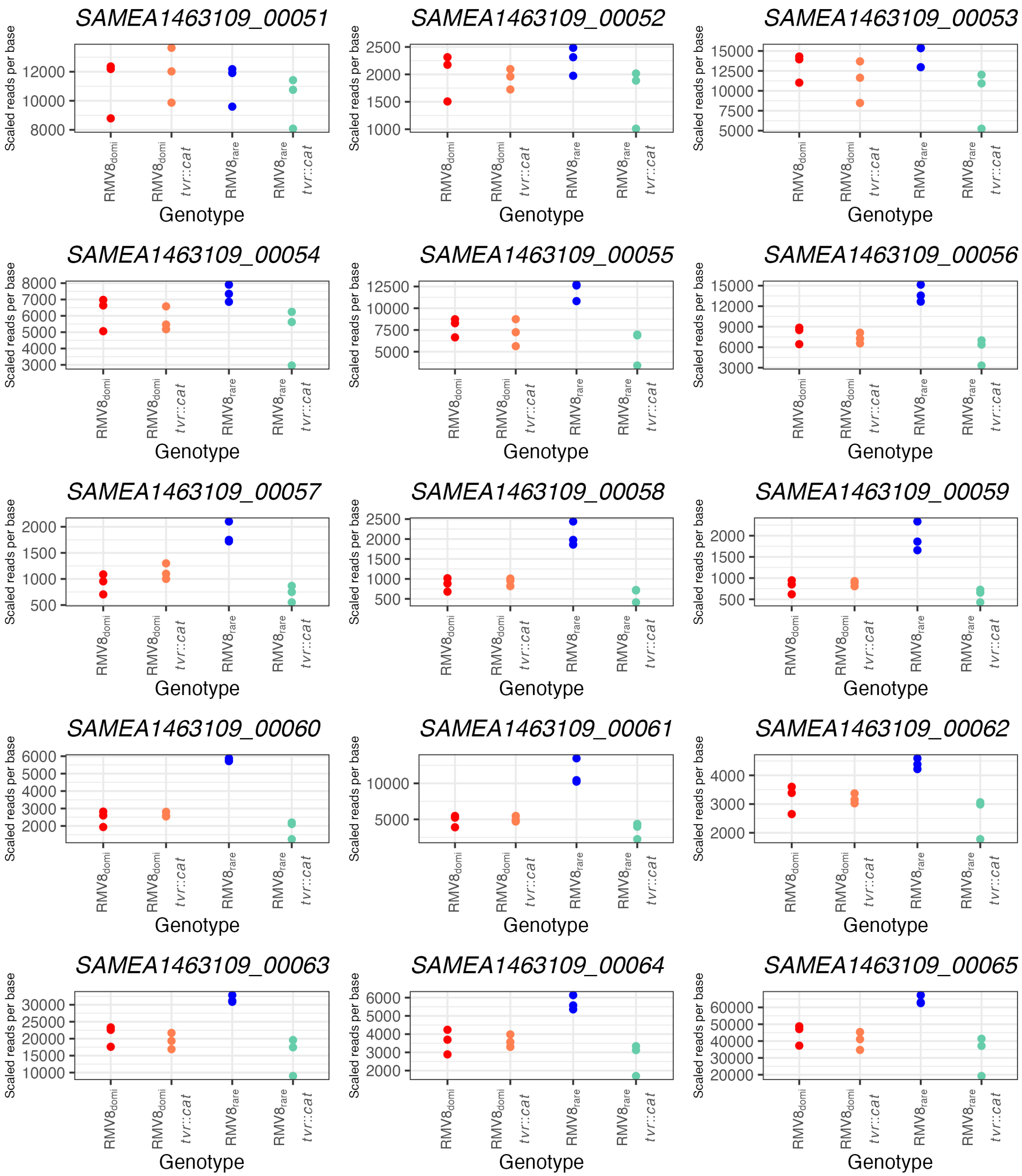

Figure S27 Quantification of the expression of ϕRMV8 structural genes using RNA-seq data. Points are coloured using the scheme described in Fig. 6. Each plot shows the transcription of a different gene, in scaled reads per base, across the four genotypes. These genes tend to be most highly expressed in RMV8$_{rare}$.

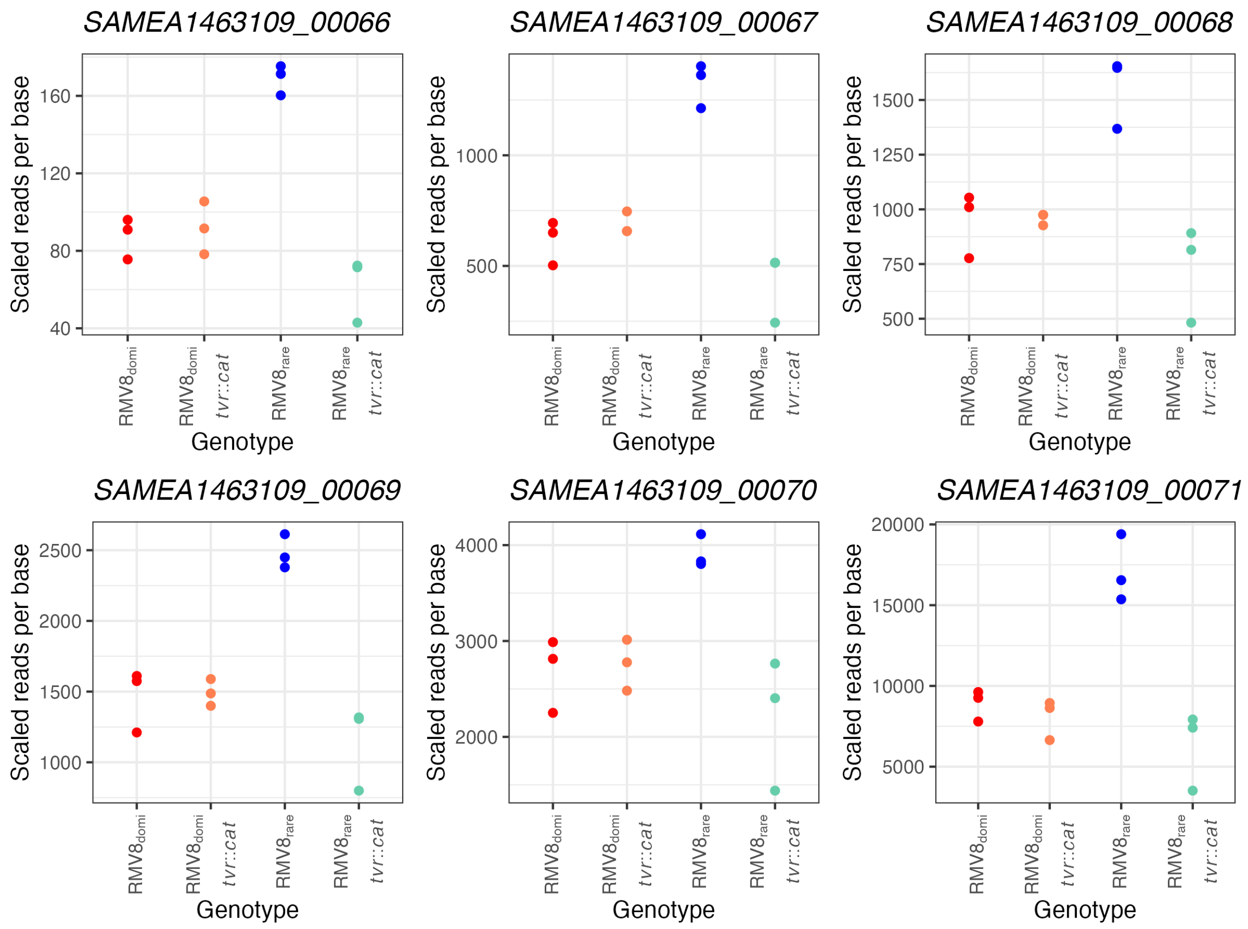

Figure S28 Quantification of the expression of ϕRMV8 lysis genes using RNA-seq data. Points are coloured using the scheme described in Fig. 6. Each plot shows the transcription of a different gene, in scaled reads per base, across the four genotypes. These genes tend to be most highly expressed in $RMV8_{rare}$.

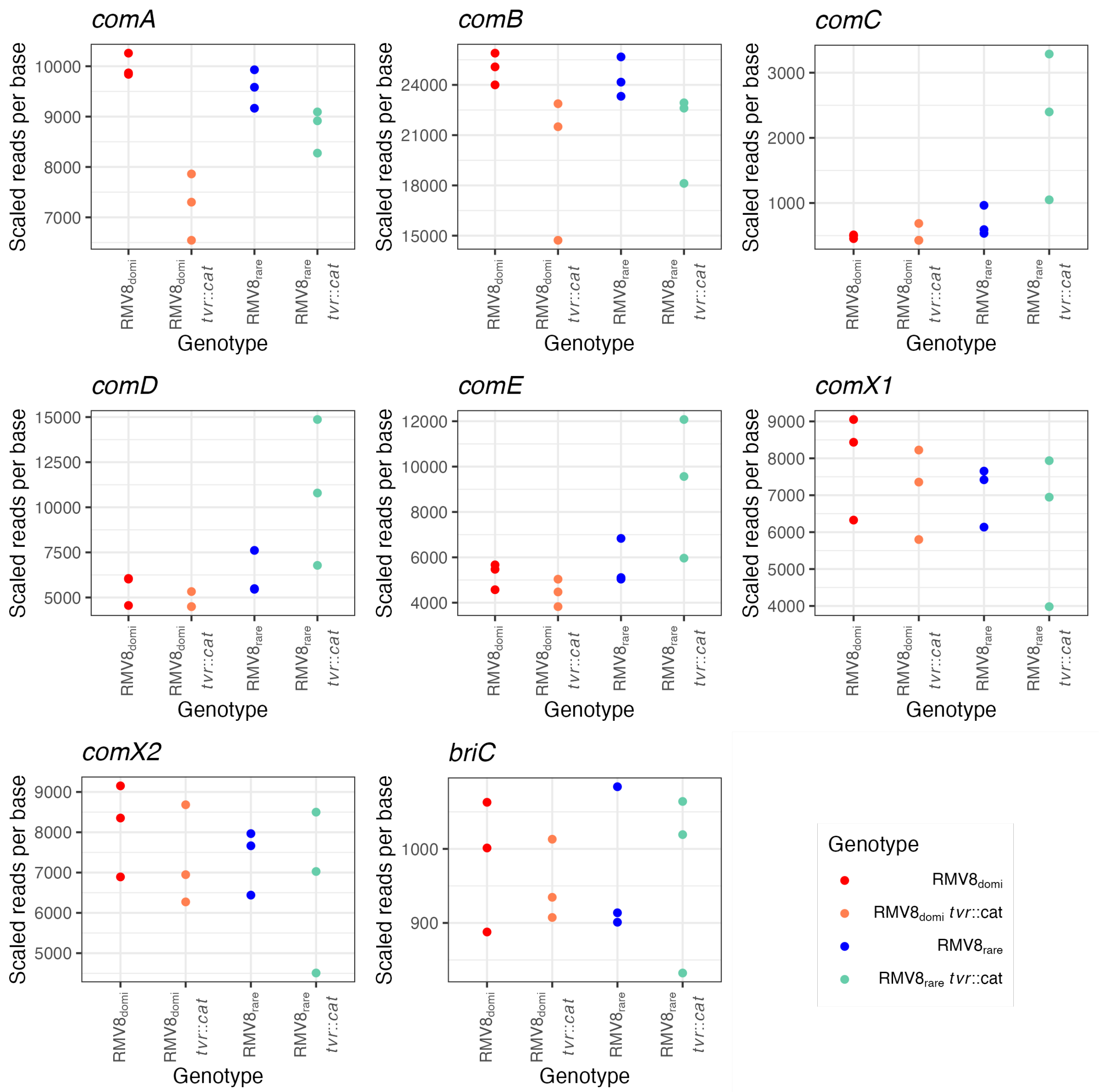

Figure S29 Quantification of the expression of early competence genes using RNA-seq data. Points are coloured using the scheme described in Fig. 6, as shown in the key. Each plot shows the transcription of a different gene, in scaled reads per base, across the four genotypes. The *comCDE* operon was more highly transcribed in RMV8$_{rare}$ *tvr*::*cat* than the other genotypes, although this difference was only significant for *comC*.

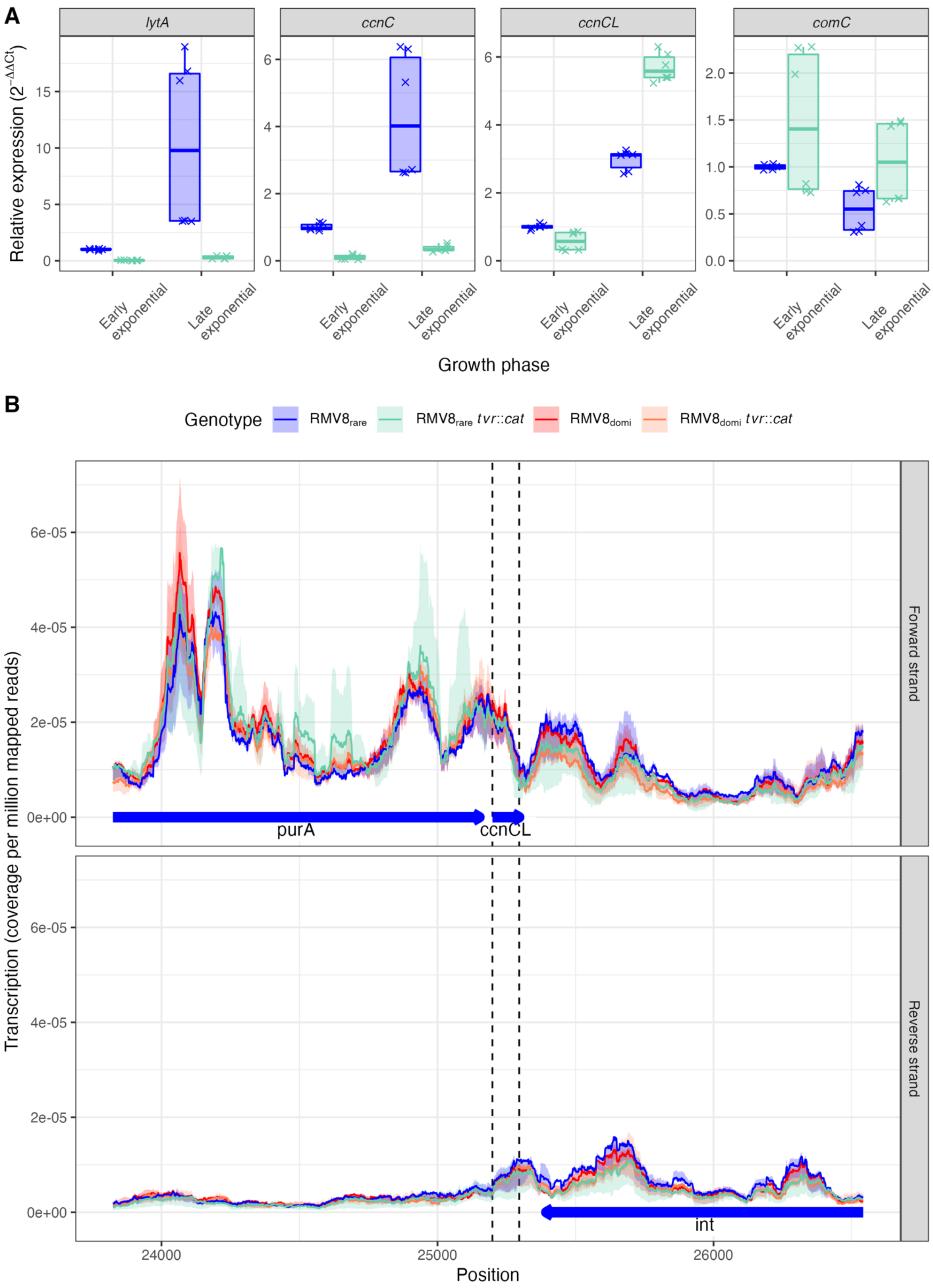
A
lytA
ccnC
ccnCL
comC
Relative expression (2^−ΔΔCt)
Early exponential
Late exponential
Growth phase
B
Genotype
RMV8rare
RMV8rare tvr::cat
RMV8domi
RMV8domi tvr::cat
Forward strand
Reverse strand
purA
ccnCL
int
Transcription (coverage per million mapped reads)
Position

Figure S30 Effects of phage activity on the expression of *ccnC*, *ccnCL* and *comC*. (A) Comparison of gene expression in $RMV8_{rare}$ and $RMV8_{rare}$ *tvr*::*cat* during the early (OD_{600} = 0.2) and late (OD_{600} = 0.5) exponential phase using qRT-PCR. Levels of transcription were quantified using the ΔΔCt approach (see Methods). Each point represents one of six measurements per gene, corresponding to three technical replicate measurements of each of two biological replicates. The interquartile range and median are summarised by the boxplots. (B) RNA-seq data at the ϕRMV8 *attL* site. The line graphs show the RNA-seq coverage, in reads per million reads mapped, of each base in the displayed *attL* region of the *S. pneumoniae* $RMV8_{rare}$ genome. The lines show the median of three replicates, and are coloured according to the genotype from which they arose. The full range of the coverage values across the replicates over all bases is shown by the shaded ribbon. The top panel shows transcription of the forward strand, with the blue arrows indicating the corresponding genes transcribed in this direction. The bottom panel shows the equivalent data for the reverse strand of the genome. The vertical dashed lines show the boundaries of the *ccnCL* gene. Quantification of the sense transcription of this locus on the forward strand by qRT-PCR is likely to be distorted by antisense transcription driven by the prophage's lysogeny genes on the reverse strand.

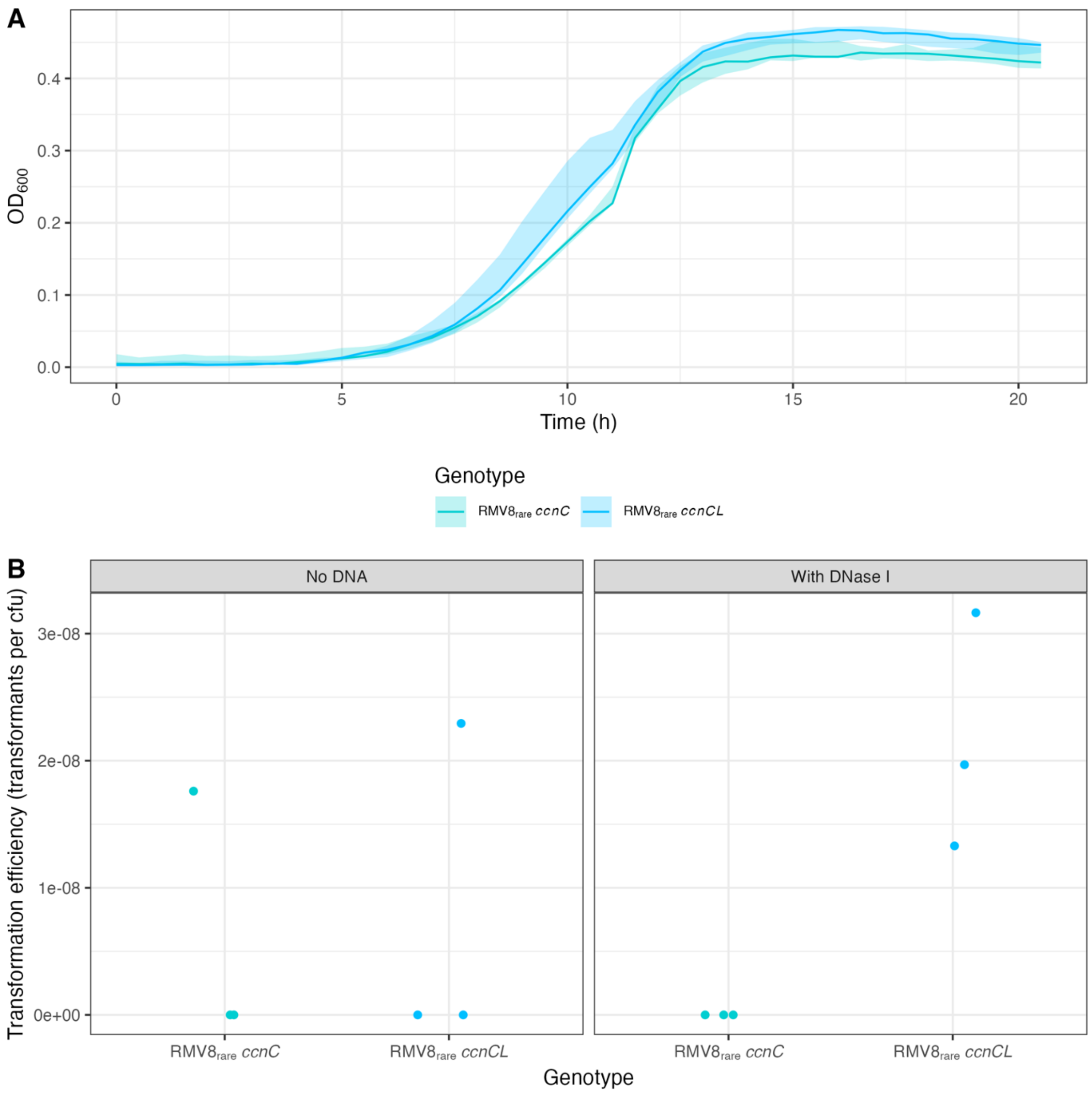

Figure S31 Characterisation of RMV8$_{rare}$ *ccnC* and *ccnCL* mutants. (A) Growth curves comparing RMV8$_{rare}$ *ccnC* and *ccnCL* mutants using three replicates. The solid line represents the median, and the ribbon shows the range between the minimum and maximum. (B) Negative controls for transformation experiments with RMV8$_{rare}$ mutants. These experiments were designed to test whether the rifampicin-resistant colonies isolated in the absence of added CSP were arising through transformation. In one set of experiments, the protocol was undertaken with no exogenous DNA. A maximum of one rifampicin-resistant colony was isolated in each experiment, likely representing the infrequent emergence of this resistance phenotype through spontaneous mutation. Similarly, the protocol was undertaken with exogenous DNA in the presence of DNase I. This again yielded few rifampicin-resistant colonies, demonstrating that the rifampicin-resistant colonies observed in the transformation experiments were primarily generated through uptake of exogenous naked DNA.
